# A genome-scale metabolic flux model of *Escherichia coli* K–12 derived from the EcoCyc database

**DOI:** 10.1186/1752-0509-8-79

**Published:** 2014-06-30

**Authors:** Daniel S Weaver, Ingrid M Keseler, Amanda Mackie, Ian T Paulsen, Peter D Karp

**Affiliations:** 1 Bioinformatics Research Group, SRI International, 333 Ravenswood Ave., 94025 Menlo Park, CA, USA; 2 Department of Chemistry and Biomolecular Science, Macquarie University, Balaclava Rd, North Ryde NSW 2109, Australia

**Keywords:** *Escherichia coli*, Flux balance analysis, Constraint-based modeling, Metabolic network reconstruction, Metabolic modeling, Genome-scale model, Gene essentiality, Systems biology, EcoCyc, Pathway Tools

## Abstract

**Background:**

Constraint-based models of *Escherichia coli* metabolic flux have played a key role in computational studies of cellular metabolism at the genome scale. We sought to develop a next-generation constraint-based *E. coli* model that achieved improved phenotypic prediction accuracy while being frequently updated and easy to use. We also sought to compare model predictions with experimental data to highlight open questions in *E. coli* biology.

**Results:**

We present EcoCyc–18.0–GEM, a genome-scale model of the *E. coli* K–12 MG1655 metabolic network. The model is automatically generated from the current state of EcoCyc using the MetaFlux software, enabling the release of multiple model updates per year. EcoCyc–18.0–GEM encompasses 1445 genes, 2286 unique metabolic reactions, and 1453 unique metabolites. We demonstrate a three-part validation of the model that breaks new ground in breadth and accuracy: (i) Comparison of simulated growth in aerobic and anaerobic glucose culture with experimental results from chemostat culture and simulation results from the *E. coli* modeling literature. (ii) Essentiality prediction for the 1445 genes represented in the model, in which EcoCyc–18.0–GEM achieves an improved accuracy of 95.2% in predicting the growth phenotype of experimental gene knockouts. (iii) Nutrient utilization predictions under 431 different media conditions, for which the model achieves an overall accuracy of 80.7%. The model’s derivation from EcoCyc enables query and visualization via the EcoCyc website, facilitating model reuse and validation by inspection. We present an extensive investigation of disagreements between EcoCyc–18.0–GEM predictions and experimental data to highlight areas of interest to *E. coli* modelers and experimentalists, including 70 incorrect predictions of gene essentiality on glucose, 80 incorrect predictions of gene essentiality on glycerol, and 83 incorrect predictions of nutrient utilization.

**Conclusion:**

Significant advantages can be derived from the combination of model organism databases and flux balance modeling represented by MetaFlux. Interpretation of the EcoCyc database as a flux balance model results in a highly accurate metabolic model and provides a rigorous consistency check for information stored in the database.

## Background

Constraint-based modeling techniques such as flux balance analysis (FBA) have become central to systems biology [1,2], enabling a wealth of informative simulations of cellular metabolism. Many constraint-based modeling techniques have been first demonstrated for the *Escherichia coli* K–12 MG1655 metabolic network. A series of *E. coli* constraint-based models have been published by the group of B. Palsson [3-6], extending work on stoichiometric constraint-based modeling of *E. coli* dating back more than twenty years [7-10]. These models constitute a gold standard for *E. coli* modeling, and have seen a range of applications [11-13] including metabolic engineering, model-driven discovery, cellular-phenotype prediction, analysis of metabolic network properties, studies of evolutionary processes, and modeling of interspecies interactions.

Motivated by the widespread use of *E. coli* metabolic models, we aimed to illustrate the benefits of integrating metabolic modeling into model organism databases by developing an *E. coli* model derived directly from the EcoCyc bioinformatics database [14]. First, we aimed to use the extensive biochemical literature referenced in EcoCyc to develop a model with improved accuracy for phenotypic prediction, specifically for predicting the phenotypes of gene knock-outs, and for predicting growth or lack thereof under different nutrient conditions.

Second, we sought to make the model easy to understand and operate. Our goal was a high level of model accessibility and readability through (a) tight web-based integration of the model with extensive model query and visualization tools, and (b) a model representation that captures extensive information that enriches the model and aids its understanding, such as metabolic pathways, chemical structures, and genetic regulatory information. Metabolic models are not just mathematical entities that output predictions; they are also artifacts that scientists interact with in multiple ways. If a model can be quickly and easily understood, scientists are more likely to trust its predictions, and the model is easier to reuse, to modify and extend, to learn from, and to validate through inspection. These aspects of a metabolic model depend strongly on how the model is represented, on the software tools available to interactively inspect the model, and on how tightly integrated the model is with those software tools.

Third, we sought to produce a model that is frequently updated to integrate new knowledge of *E. coli* metabolism.

Fourth, we sought to use the EcoCyc-derived *E. coli* metabolic model to identify errors in EcoCyc, and open problems in *E. coli* biology, by performing in-depth investigations of the disagreements between the phenotypic predictions of the model and experimental results.

We present EcoCyc–18.0–GEM, a constraint-based genome-scale metabolic model for *E. coli* K–12 MG1655 that is directly derived from the EcoCyc model organism database (http://EcoCyc.org) built on the genome sequence of *E. coli* K–12 MG1655. The model is implemented using the MetaFlux [15] component of the Pathway Tools software [16].

## Results and discussion

The EcoCyc–18.0–GEM generated from EcoCyc 18.0 encompasses 1445 genes, 2286 unique cytosolic and periplasmic reactions, and 1453 unique metabolites. Table 1 compares the statistics of EcoCyc–18.0–GEM with previous *E. coli* metabolic models. EcoCyc–18.0–GEM is an advance over previous stoichiometric models of *E. coli* metabolism in four respects: in its size; in its accuracy; in its form, readability and accessibility; and in its update frequency. Here we summarize these results; these points will be expanded in subsequent subsections.

**Table 1:** **Survey of recent *E. coli *** **genome-scale model statistics**

**Statistics**	**Feist *et al.*** [5]	**Orth *et al.*** [6]	**EcoCyc–18.0–GEM**
	**2007**	**2011**	
# Genes	1260	1366	1445
# Unique reactions	1721	1863	2286
# Unique metabolites	1039	1136	1453
Gene knockout accuracy	91.4%	91.3%	95.2%
# PM growth conditions	170	–	431
PM growth condition	75.9%	–	80.7%
accuracy			
# biomass metabolites	65	72	108

Gene knockout prediction accuracy represents simulated growth on glucose minimal media under aerobic conditions. Reaction and metabolite counts represent reactions found in the cytosol and periplasm, since EcoCyc–18.0–GEM does not cover porin-mediated diffusion of metabolites into the periplasmic space.

The MetaFlux component of Pathway Tools translates Pathway/Genome Database (PGDB) reactions and compounds into constraint-based metabolic models. Our methodology of fusing systems-biology models and bioinformatics databases has several advantages because of strong synergies between these approaches. Databases and models both require extensive literature-based curation and refinement. It is more efficient to perform that curation once in a manner that benefits a database and a model, than to duplicate curation efforts for a database project and a modeling project. Furthermore, the modeling process identifies errors, omissions, and inconsistencies in the description of a metabolic model, and therefore drives correction and further curation of the database if the two efforts are coupled. We made more than 80 EcoCyc updates as a result of comparing model predictions with experimental data and literature for this work. In addition, bioinformatics database curation methods such as the use of evidence codes and citations to provide data provenance, and the incorporation of mini-review summaries that describe enzymes and pathways, benefit systems-biology models, which typically lack data provenance and explanations.

### 

#### 

##### 

**Advances in model size.** Compared with iJO1366, EcoCyc–18.0–GEM represents a 6% increase in the number of genes, a 23% increase in the number of unique cytosolic and periplasmic reactions, and a 28% increase in the number of unique metabolites. The size of EcoCyc–18.0–GEM is currently exceeded only by the more mathematically complex ME-model of O’Brien *et al.*[17], which includes simulation of gene expression, transcriptional regulation, and protein synthesis.

##### 

**Advances in model accuracy.** We conducted a threephase validation of EcoCyc–18.0–GEM to assess its accuracy (see Table 1). In phase I we compared simulated EcoCyc–18.0–GEM rates of nutrient uptake and product secretion in aerobic and anaerobic glucose culture with experimental rates derived from chemostat culture; the performance of EcoCyc–18.0–GEM was equivalent to previous models. In phase II we compared essentiality prediction for all 1445 genes involved in the model with experimental gene essentiality datasets; its error rate in predicting gene-knockout phenotypes decreased by 46% over the best previous model. In phase III we compared nutrient utilization predictions of EcoCyc–18.0–GEM with 431 experimental nutrient utilization tests; its accuracy in predicting growth and respiration under different nutrient conditions increased by 4.8% over previous models as the number of nutrient conditions expanded 2.5-fold. We investigated conflicts between experimental results and predictions of EcoCyc–18.0–GEM in detail, and provide an extensive discussion of these conflicts within the context of EcoCyc and the literature.

Subjects of particular interest include alternative catalytic routes capable of replacing genes thought to be essential; compounds with unclear routes of catabolism which are capable of supporting growth and/or cellular respiration; regulatory and environmental perturbations of the stoichiometric network model; and investigations of what, exactly, constitutes gene essentiality.

##### 

**Advances in model form, readability, and accessibility.** Another benefit of coupling systems-biology models with databases, and a corresponding advance of our model, is that generating a constraint-based model from a database that has associated web-based visualization tools leads to a *literate model* (by analogy to Knuth’s notion of literate programming [18]). A literate model is easy to read, and is highly accessible to and understandable by scientists.

##### 

**Advances in model update frequency.** Because the MetaFlux component of Pathway Tools generates constraint-based models directly from the EcoCyc PGDBs, as the database is refined through new curation, those refinements are automatically incorporated into newly generated versions of the model. We release new versions of the EcoCyc-based model three times per year; previous models were updated every four years [4-6]. Although there are reasons to limit the frequency of releases in order to tie them to a well-defined version of the database and throughly test the accuracy of new versions, we believe that more frequent model updates are useful for an organism as important as *E. coli*.

### Validation of biomass metabolites, nutrients, and secretions

Refinement of EcoCyc–18.0–GEM began with the validation of the biomass, nutrient, and secretion metabolite sets, which are detailed at length in Additional file 1 and Additional file 2: Table S1. The biomass metabolite set establishes requirements for growth and determines the growth rate of the simulation. The biomass metabolite set for EcoCyc–18.0–GEM was based on the iJO1366 wild-type and core biomass reaction sets published by Orth *et al.*, with several revisions stemming from differences in content and functionality between EcoCyc and the iJO1366 model. Gene essentiality in constraint-based models is principally determined by the biomass demands of the cell. Inclusion of a metabolite in the biomass metabolite set forces the genes required for manufacture of that metabolite to become essential in the simulation.

A wild-type biomass metabolite set, which is derived from measurement of the biomolecular composition of healthy, growing cells, is not representative of the minimal set of biomass metabolites required for cell survival. Because biomass metabolites not truly required for cell survival will generate false simulation predictions of essentiality in their biosynthetic pathways, the concept of a core biomass metabolite set was developed by Feist *et al.* The core biomass metabolite set is a biomass metabolite set that is defined with the aim of maintaining quantitative accuracy with regards to cell performance while predicting the observed experimental essentiality data as accurately as possible. Because much of this work focuses on testing the minimum requirements for cell growth, we frequently employed the core biomass metabolite set in our simulations. We use the term “expanded biomass set” to refer to our version of the wild-type biomass metabolite set described in Orth *et al.*, because we do not wish to imply that the simulated cells always represented the wild-type state.

The biomass metabolite sets described here underwent several revisions reflecting differences in scope between EcoCyc–18.0–GEM and iJO1366. Whereas iJO1366 is a purpose-built model developed using the COBRA Toolbox with input from KEGG, EcoCyc, and other databases, EcoCyc is a database with its own schema whose entries are programmatically transformed into an FBA model. The specific metabolites present in iJO1366 therefore cannot always be matched with the specific metabolites generated from EcoCyc by MetaFlux on a one-to-one basis. Several biomass metabolites represented as distinct within iJO1366, such as phosphatidylethanolamines with different chain lengths and saturations, are summed under the heading of a single representative metabolite in the EcoCyc–18.0–GEM biomass set. Additionally, not all processes covered in EcoCyc–18.0–GEM are covered in iJO1366, and the reverse is also true. As a result, the biomass metabolite sets differ slightly. Additional file 2: Table S2 contains a complete side-by-side comparison of the EcoCyc–18.0–GEM and iJO1366 biomass metabolite sets, and lists the differences between them.

We constructed standard nutrient sets based on culture conditions reflecting experiments in glucose or glycerol minimal media and on the model’s capability to use substrates. Those substrates include glucose or glycerol as appropriate, O _2_, $NH_{4}^{+}$, phosphate, sulfate, ferrous iron, water, CO _2_, minerals appropriate to the biomass objective function, and MOPS buffer (usable as a sulfur source) where appropriate. Because of passive diffusion at the high concentration of ammonium used in experimental culture, $NH_{4}^{+}$ is supplied directly in the cytosol (avoiding false negative essentiality predictions for the high-affinity nitrogen transporter *amtB*), whereas all other nutrients are supplied in the periplasmic space.

Finally, we developed a large set of secreted compounds that could be supplied across all growth conditions explored with our model. It contains both plausible products of *E. coli* metabolism and dead-end metabolites [19] within the model. The presence or absence of metabolites in this set should not be construed to indicate their presence or absence in *E. coli* culture media.

We verified the metabolic reachability of each component within the EcoCyc biomass metabolite set by supplying nutrients representing an aerobic glucose minimal medium and setting the production of each individual metabolite in turn as the optimization goal of MetaFlux, and repaired gaps by means of literature-based manual curation of EcoCyc and expansion of the relevant metabolite sets.

### ATP maximization validation

We next confirmed that simulations of aerobic growth on glucose run with maximization of ATP production as their objective made appropriate use of the glycolytic and TCA cycle pathways and agreed with previous work on *E. coli* FBA.

The maximization of ATP production under aerobic conditions was studied by setting the ATP consumption reaction ATP + H _2_O → ADP + Pi + H ^+^ as the objective function to be maximized by MetaFlux. The fluxes resulting from the maximization of ATP production on glucose under aerobic conditions were compared with fluxes from COBRA Toolbox [20] simulations of iJO1366 under the same conditions and were found to be largely identical (Table 2). Differences arose from variances in proton translocation stoichiometries between the EcoCyc–18.0–GEM version of the NADH:ubiquinone oxidoreductase I (NADH-DEHYDROG-A-RXN) (4 H ^+^ translocated per 2 e ^-^, as proposed by Treberg *et al.*[21]) and the iJO1366 version of the NADH:ubiquinone oxidoreductase (NADH16pp) (3 H ^+^ translocated per 2 e ^-^, as proposed by Wikstrom and Hummer [22]). The exact number of protons translocated by the NADH:ubiquinone oxidoreductase is an issue of open discussion in the scientific literature, and this uncertainty is described in the EcoCyc summary for the enzyme. If a consensus develops behind the 3 H ^+^ per 2 e ^-^ view of translocation stoichiometry, future versions of EcoCyc will be changed to reflect this fact.

**Table 2:** Flux comparison between EcoCyc–18.0–GEM and iJO1366: ATP maximization objective under aerobic conditions

**EcoCyc**		**iJO1366**	
**Reaction**	**Flux**	**Reaction**	**Flux**
ATP maximization objective	216	ATPM	235
Glucose uptake	10	EX_glc(e)	10
O _2_ uptake	60	EX_o2(e)	60
H _2_O production	60	EX_h2o(e)	60
CO _2_ production	60	EX_co2(e)	60
ATP synthase*	176	ATPS4rpp	195
NADH-DEHYDROG-A-RXN	100	NADH16pp	100
Cytochrome *bo* oxidase*	60	CYTBO3_4pp	120
PYRNUTRANSHYDROGEN-RXN	20	NADTRHD	20
GAPOXNPHOSPHN-RXN	20	GAPD	20
ISOCITDEH-RXN	20	ICDHyr	20
3PGAREARR-RXN	20	ENO	20
ACONITATEDEHYDR-RXN	20	ACONTa/b	20
PHOSGLYPHOS-RXN	20	PGK	20
CITSYN-RXN	20	CS	20
2OXOGLUTARATEDEH-RXN	20	AKGDH	20
2PGADEHYDRAT-RXN	20	ENO	20
Succinate dehydrogenase*	20	SUCDi	20
MALATE-DEH-RXN	20	MDH	20
FUMHYDR-RXN	20	FUM	20
PYRUVDEH-RXN	20	PDH	20
SUCCOASYN-RXN	20	SUCOAS	20
PGLUCISOM-RXN	10	PGI	10
Glucose PTS uptake*	10	GLCptspp	10
TRIOSEPISOMERIZATION-RXN	10	TPI	10
RXN0-313	10	F6PA	10
2.7.1.121-RXN	10	DHAPT	10

Glucose uptake is set to 10 mmol/gCDW/hr. Other nutrient and secretion fluxes are unbounded. Flux rates are reported as absolute values for clarity. True iJO1366 fluxes for EX_glc(e), EX_o2(e), PGK, PGM, and SUCOAS are negative because of reaction directionality convention. Most EcoCyc reactions are identifed by their EcoCyc frame IDs. The remainder are marked with asterisks, and their frame IDs are as follows: ATP synthase EcoCyc frame ID: TRANS-RXN-249; cytochrome *bo* oxidase EcoCyc frame ID: RXN0-5268; succinate dehydrogenase EcoCyc frame ID: SUCCINATE-DEHYDROGENASE-UBIQUINONE-RXN; glucose PTS uptake EcoCyc frame ID: TRANS-RXN-157/RXN0-6717/RXN0-6718; fructose 6-phosphate aldolase EcoCyc frame ID: RXN0-313; dihydroxyacetone kinase EcoCyc frame ID: 2.7.1.121-RXN.

Further numerical differences are due to a technical consideration: EcoCyc cytochrome *bo* oxidase reaction stoichiometry is written in terms of whole molecules of oxygen, while iJO1366 CYTBO3_4pp is written in terms of half-molecules (1 O _2_ consumed vs. 0.5 O _2_).

### Comparison with iJO1366 simulation and chemostat data

After completing our basic validation of biomass production and energy generation, we maximized the rate of EcoCyc–18.0–GEM biomass metabolite set production under several minimal media conditions and ensured that we obtained results comparable to the iJO1366 results for the same conditions obtained using the COBRA Toolbox. Divergences were addressed by literature-based manual curation of EcoCyc and modification of MetaFlux reaction sets. We further compared the extracellular flux distributions resulting from these simulations with the experimental data obtained in carbon-limited chemostat environments under both aerobic and anaerobic conditions.

Tables 3 and 4 compare extracellular metabolite flux results derived from EcoCyc–18.0–GEM simulation, iJO1366 simulation, and experimental data [23,24] for the canonical cases of aerobic and anaerobic growth on glucose-limited chemostat culture. In all simulations, the experimental rate of glucose supply was the only fixed constraint; all other nutrients and secretions were left unconstrained.

**Table 3:** Comparison of experimental aerobic glucose-limited chemostat growth data with EcoCyc–18.0–GEM and iJO1366 constraint-based model predictions

**Aerobic fluxes**	**Experimental**	**EcoCyc**	**iJO1366**
Specific growth rate (*µ*) (1/hr)	0.300	0.272	0.288
Glucose uptake (*mmmol*/*g*_*CDW*_/*hr*)	3.008	3.008	3.008
O _2_ uptake	7.413	6.158	5.703
NH _4_ uptake	2.367	2.899	3.021
Sulfate uptake		0.067	0.073
Phosphate uptake		0.260	0.267
CO _2_ production	7.38	6.850	6.288
H _2_O production		13.926	13.704
H ^+^ production		2.454	2.552

Metabolite uptake and production rates are in units of mmol/gCDW/hr. Growth is in units of hr ^-1^. Experimental data from Kayser *et al.*[23].

**Table 4:** Comparison of experimental anaerobic glucose-limited chemostat growth data with EcoCyc–18.0–GEM and iJO1366 constraint-based model predictions

**Anaerobic fluxes**	**Experimental**	**EcoCyc**	**iJO1366**
Specific growth rate *µ* (*hr*^-1^)	0.30	0.24	0.24
Glucose uptake (*mmol*/*g*_*CDW*_/*hr*)	10.0	10.00	10.00
O _2_ uptake	0.00	0.00	0.00
NH _4_ uptake		2.52	0.82
Sulfate uptake		0.06	0.06
Phosphate uptake		0.23	0.22
CO _2_ production		0.04	-0.08
H _2_O production		-1.92	-1.84
H ^+^ production	27.8	27.5	
Acetate production	7.5	8.29	8.23
Formate production	11.3	17.37	17.25
Succinate production	1.2	0.00	0.08
Ethanol production	8.7	8.13	8.08

Metabolite uptake and production rates are in units of mmol/gCDW/hr. Growth is in units of hr ^-1^. Experimental data from [24] via [25]. Formate-hydrogen lyase (FHLMULTI-RXN) was left inactive for purposes of comparison.

**Figure 1:**
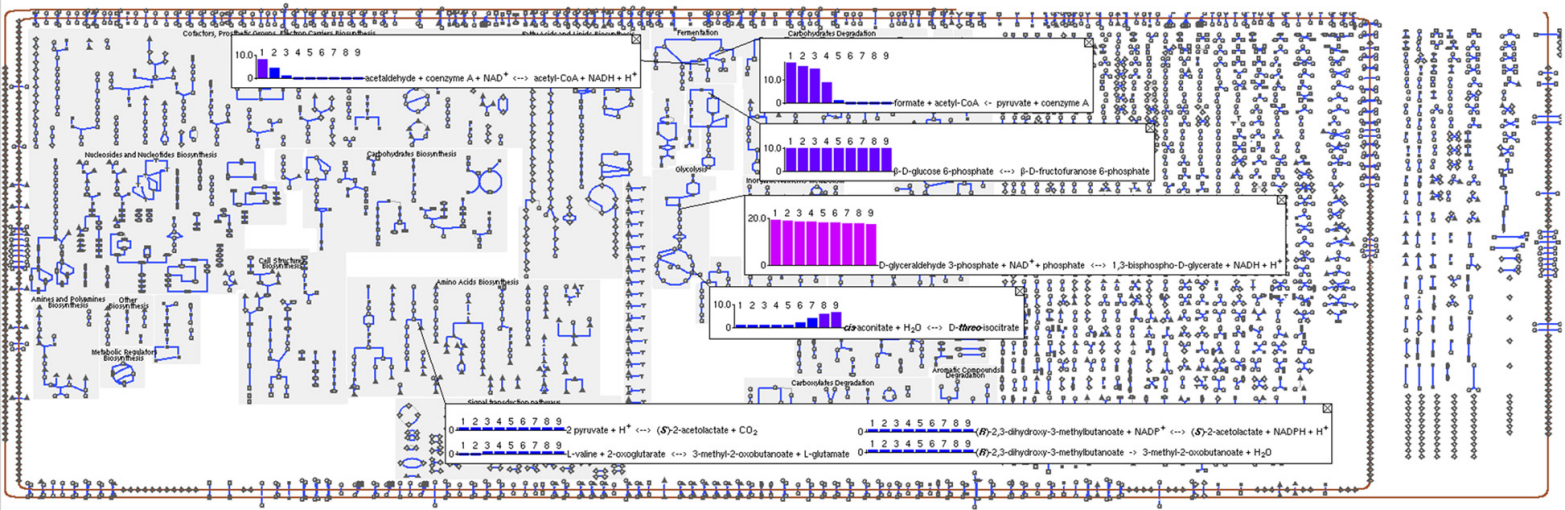
**Pathway Tools visualization of EcoCyc–18.0–GEM flux during aerobic transition.** Example visualization of EcoCyc–18.0–GEM flux during a transition from anaerobic to aerobic growth, created within the interactive Cellular Overview diagram in Pathway Tools. The upper bound of glucose uptake is set to 10 mmol/gCDW/hr, while the upper bound of oxygen uptake is increased from 0 to 20 mmol/gCDW/hr in 2.5 mmol/gCDW/hr steps. Omics Popups are used to illustrate flux through acetaldehyde dehydrogenase, pyruvate-formate lyase, phosphoglucose isomerase, glyceraldehyde 3-phosphate dehydrogenase, cis-aconitate hydratase, and valine biosynthesis.

**Figure 2:**
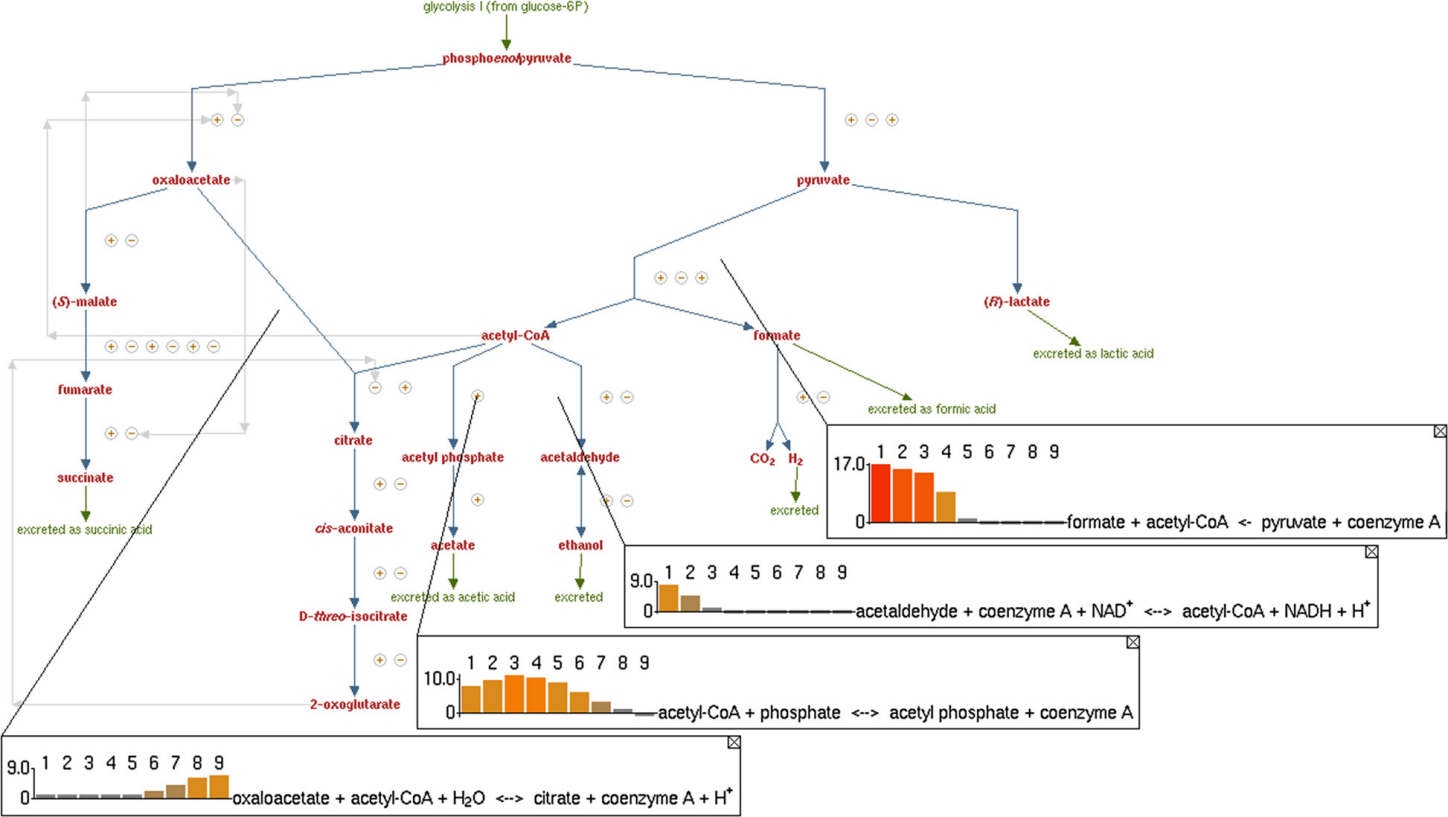
**Pathway Tools visualization of mixed-acid fermentation flux during aerobic transition.** Visualization of EcoCyc–18.0–GEM flux in mixed-acid fermentation during a transition from anaerobic to aerobic growth, created within the EcoCyc mixed-acid fermentation pathway page in Pathway Tools. The upper bound of glucose uptake is set to 10 mmol/gCDW/hr, while the upper bound of oxygen uptake is increased from 0 to 20 mmol/gCDW/hr in 2.5 mmol/gCDW/hr steps. Omics Popups are used to illustrate changes in flux to the mixed-acid fermentation products formate, acetate, and ethanol as the cellular energy and redox balance evolves during the aerobic transition.

The behavior of MetaFlux/EcoCyc–18.0–GEM simulations was very similar in most regards to the behavior of COBRA/iJO1366 simulations. Respiration and fermentation rates scaled with nutrient uptake at comparable rates. The generally higher rates of O _2_ uptake observed experimentally lend support to a lower practical efficiency of proton translation stoichiometry *in vivo*, perhaps augmented by respiratory inefficiencies such as futile cycling and generation of reactive oxygen species. Both models secrete the expected 1:2:1 mix of acetate, formate, and ethanol during anaerobic growth on glucose that Varma *et al.*[25] originally identified as stoichiometrically optimal. During the transition between purely anaerobic and aerobic domains, the competing demands of energy metabolism and redox elimination cause a characteristic pattern of mixed acid fermentation described by Varma *et al.*, in which ethanol, then formate, and finally acetate production are eliminated as the cell’s oxygen supply becomes completely sufficient to support aerobic respiration. Figures 1 and 2 use the Cellular Overview and Omics Popup visualization functionalities of Pathway Tools to illustrate this behavior in EcoCyc–18.0–GEM during a transition from anaerobicity to aerobicity.

Comparisons between FBA-predicted extracellular fluxes and experimental fluxes show that EcoCyc–18.0–GEM and iJO1366 FBA predictions agree more closely with each other than with experimental flux results, although the correspondence between simulation and experiment was quite close for the experimental fluxes under consideration. This result was expected given the adaptation of the iJO1366 biomass function for use in EcoCyc–18.0–GEM, the use of iJO1366 and preceding reconstructions as benchmarks in the development of EcoCyc–18.0–GEM, and the use of EcoCyc as a reference in the construction of iJO1366 and its predecessors. The experimental measurements generally demonstrate higher fluxes of the respiratory gases O _2_ and CO _2_ than the simulated fluxes, suggesting a degree of respiratory inefficiency not properly modeled by FBA. Similarly, small quantities of succinate and lactate were produced by experimental fermentation, indicating a degree of divergence from metabolic optimality *in vivo*. Broader cellular constraints such as regulation, protein crowding, pathway enzyme synthesis requirements, and pathway-throughput limits underlie these differences [17,26,27]. Successive generations of evolution under constant growth conditions might bring the experimental result closer to theory, as described in Ibarra *et al.*[28].

### Gene essentiality analysis

One of the most exciting aspects of genome-scale flux modeling is the ability to rapidly test computational gene knockouts (KOs) for their effects on metabolic function. Gene KO simulation is useful both for prediction and for validation: *in silico* FBA screens of gene KOs have been applied in a variety of metabolic engineering efforts [29-31], and *E. coli* KO library collections with well-characterized growth behavior provide an important tool for flux model validation.

FBA gene KO essentiality prediction depends on two types of database associations between genes and chemical reactions: genes whose products catalyze reactions, and genes whose products are reaction substrates (e.g., acyl-carrier protein). Simulation gene KOs are carried out by identifying all reactions involving the gene, and then identifying all other genes capable of catalyzing the reactions or supplying the substrates thus identified. Reactions for which no isozymes or alternative substrate supplies are found are removed from the FBA stoichiometric network. An FBA solution is then calculated for the new model. If the simulated gene KO has caused the deletion of one or more reactions required for the synthesis of a biomass metabolite, generation of the full biomass metabolite set will be blocked and the FBA simulation returns a no-growth result. Such a result represents a prediction of gene essentiality. If the complete biomass metabolite set can still be produced in spite of the simulated gene knockout, the FBA simulation returns a growth result, indicating a prediction of gene non-essentiality.

The experimental essentiality data used in our tests consisted of two major datasets. The first, used to study gene essentiality on rich and glucose minimal media, was the deletion study of Baba *et al.*[32] as updated by Yamamoto *et al.*[33], which tested the Keio collection library of 4288 *E. coli* gene deletion strains for growth on LB rich media and MOPS minimal media with 0.4% glucose. We conducted our glycerol minimal media tests using the gene knockout essentiality data of Joyce *et al.*[34], an expansion of the study of the Keio collection essentiality to include growth on M9 minimal medium with 1% glycerol.

Several *E. coli* gene deletions strongly affect growth on various types of minimal media, but are nonessential to growth on rich media. Because the FBA simulation result is treated as a binary test (growth or no-growth), gene deletions that strongly affect growth on minimal media without producing a completely lethal phenotype must be defined either as experimentally essential or as experimentally nonessential.

Two representative perspectives on this definition are the narrow essentiality criteria of no observable growth in minimal media and the broad essentiality criteria used by Orth *et al.* The narrow glucose essentiality criteria treat as essential those Baba *et al.* and Yamamoto *et al.* gene deletion mutants with OD600 ≤ 0.005 after 24 and 48 hr growth on glucose minimal media. This requires no perceptible growth over a long period. The broad essentiality criteria was originally defined in relative terms by Joyce *et al.*, as the slowest-growing ninth of all Keio collection deletion mutants.

**Figure 3:**
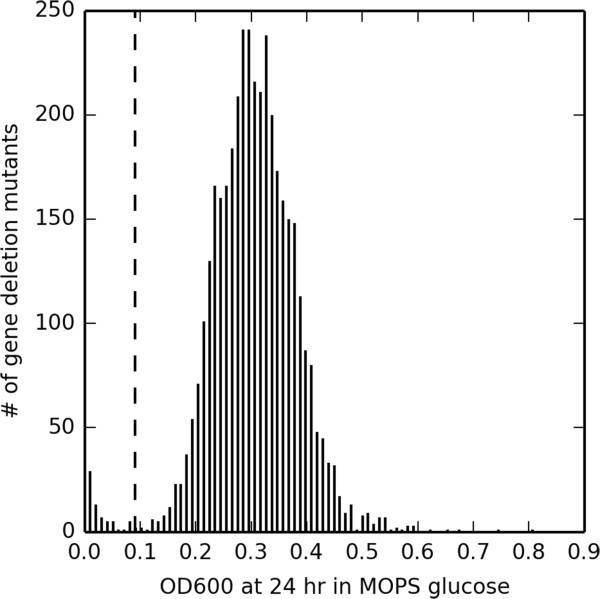
**Essentiality criteria basis in high-throughput KO data.** Histogram of OD600 measurements for all rich media-viable Baba *et al.* deletion mutants after 24 hr of growth on MOPS media containing 0.4% glucose. Data from Supplementary Table three of Baba *et al*.

In absolute terms, that approach treats as essential those deletion mutants measured by Baba *et al.* to have OD ≤ 0.091 after 24 hr growth on glucose minimal media, which indicates impaired growth over a shorter period. The practical difference between these two perspectives is illustrated in Figure 3, which displays the distribution of OD600 data for all rich media-viable Keio collection mutants after 24 hr of growth on MOPS media containing 0.4% glucose, as originally published in Supplementary Table three of Baba *et al.* As the figure illustrates, the broad essentiality criteria include a population of cells with severe growth defects that is not contained in the narrow essentiality data. The comparison between narrow and broad essentiality criteria can be expanded to glycerol minimal media by comparing narrow glycerol essentiality criteria of no observed growth on rich media with the glycerol essentiality criteria of Orth *et al.*, again derived from the criteria of Joyce *et al.* involving successive division into thirds.

In order to examine criteria for experimental gene essentiality more deeply and to illustrate the effect of defining a core biomass metabolite set, we conducted essentiality testing using both the expanded and core biomass metabolite sets proposed by Orth *et al.* Differences in essentiality predictions between the two data sets illustrated the differences between standard cell composition under nominal conditions and the minimal composition required for cell growth.

We simulated single gene KOs on glucose and glycerol minimal media for the 1445 genes in EcoCyc–18.0–GEM to test whether the resulting EcoCyc–18.0–GEM gene deletion mutants were capable of generating core and expanded biomass metabolite sets from sets of nutrients based on the experimental culture media of Baba *et al.* and Joyce *et al.* Gene KO simulations capable of generating any growth at all were scored as nonessential, whereas gene KOs blocking generation of the biomass metabolite set were scored as essential.

We compared the results of this simulated essentiality screen with experimental gene essentiality results based on both narrow and broad gene essentiality criteria. Incorrect essentiality predictions were addressed by literature-based manual curation of EcoCyc and modification of MetaFlux metabolite sets. Final essentiality prediction results after curation are summarized in Tables 5 for glucose and 6 for glycerol. The overall accuracy of prediction for growth on glucose with the core biomass metabolite set and broad essentiality criteria was 1375/1445 (95.2% accuracy, 99.0% sensitivity, 77.5% specificity). For prediction of growth on glycerol under the same simulation conditions, the overall accuracy of prediction was 1365/1445 (94.5% accuracy, 98.1% sensitivity, 77.5% specificity). Sensitivity here refers to the percentage of gene deletions resulting in growth that are correctly identified by simulation, while specificity refers to the percentage of gene deletions resulting in no growth that are correctly identified by simulation.

**Table 5:** Comparison of experimental gene essentiality results with computational EcoCyc–18.0–GEM results for aerobic growth on MOPS medium with 0.4% glucose

**KO growth on glucose (sim/exp)**	**BC**	**BE**	**NC**	**NE**
True positive (growth/growth)	1175	1136	1204	1164
False positive (growth/no growth)	58	64	29	36
False negative (no growth/growth)	12	51	78	118
True negative (no growth/no growth)	200	194	134	127

Four comparisons are provided — gene essentiality is evaluated with regard to (B)road and (N)arrow experimental essentiality criteria, and with regard to a (C)ore biomass metabolite set that maximizes the accuracy of essentiality predictions as well as an (E)xpanded biomass metabolite set reflecting experimental measurements of healthy cells. The results of these comparisons are arranged based on correspondence between simulation and experiment: (1) true positive (simulation predicts growth, experiment shows growth), (2) false positive (simulation predicts growth, experiment shows no respiration), (3) false negative (no simulated growth, but experiment shows respiration), and (4) true negative (no simulated growth, experiment shows no respiration).

**Table 6:** Comparison of experimental gene essentiality results with computational EcoCyc–18.0–GEM results for aerobic growth on MOPS medium with 1% glycerol

**KO growth on glycerol (sim/exp)**	**BC**	**BE**	**NC**	**NE**
True positive (growth/growth)	1165	1124	1195	1154
False positive (growth/no growth)	58	63	28	35
False negative (no growth/growth)	22	63	102	143
True negative (no growth/no growth)	200	195	120	113

See Table 5 caption for description of column headings.

Tables 5 and 6 illustrate that the gene essentiality predictions in EcoCyc–18.0–GEM differed in a number of cases from the gene essentiality conclusions generated by high-throughput gene KO screening. Because these are situations of considerable interest to the development of EcoCyc as a reference, we examined them in greater detail for the case of growth on glucose, with reference to the *E. coli* literature. Our examination covered two types of incorrect gene deletion growth predictions. The first type was a false positive growth prediction. These genes, which are experimentally essential under the conditions tested by Baba *et al.*, were predicted to be nonessential by EcoCyc–18.0–GEM. The second type was a false negative growth prediction. These genes, which are not experimentally essential under the conditions tested by Baba *et al.*, were predicted to be essential by EcoCyc–18.0–GEM.

Tables 7, 8, 9, 10, 11 and 12 present five broad categories of incorrect gene deletion predictions from EcoCyc–18.0–GEM. Table 7 cover false predictions involving open questions of *E. coli* biology, false predictions resulting from interesting facets of experimental or simulation methods, and other situations of special relevance. Table 8 covers false predictions in core glycolytic, pentose phosphate, Entner-Doudoroff, and TCA cycle metabolism. This highly interconnected region of *E. coli* metabolism contains several isozymes and opportunities for reversibility, and presents a challenge to FBA essentiality predictions in the absence of complete regulatory modeling. Table 9 cover false predictions that are the result of unmodeled regulation of gene expression or enzyme activity. Genes repressed under Baba *et al.* experimental growth conditions, insufficiently expressed isozymes, and cases of enzyme inhibition all fall into this category. Table 10 covers situations in which the essentiality conclusions of the high-throughput essentiality screen differed significantly from the essentiality conclusions made by the *E. coli* K–12 literature. Table 11 covers false gene essentiality predictions relating to systems beyond the scope of EcoCyc–18.0–GEM’s biomass objective function. Finally, Table 12 covers false gene essentiality predictions made as a result of MetaFlux and EcoCyc technical problems discovered in the course of this study.

**Table 7:** **False gene essentiality predictions resulting from open questions in *E. coli * biology and gene essentiality**

**Gene**	**HT**	**Sim**	**Conv**	**Citations**	**Comments**
*argD*	+	–	+	[35,36]	EcoCyc lists *argD* as the only enzyme capable of carrying out the N-succinyldiaminopimelate aminotransferase reaction in lysine biosynthesis. Cox and Wang [35] demonstrate that a second, thus far unidentified enzyme besides ArgD can catalyze the DapC reaction. *argD* is discussed in [36].
*cysC*	–	+	–	[5,32,34,37,38]	Gene essentiality in the sulfate utilization pathway is bypassed until *cysIJ* in the experiments of [32],
*cysH*	–	+	–		which were conducted in a MOPS buffer capable of being used as a sulfur source via *ssuEADCB*
*cysN*	–	+	+		alkanesulfonate desulfonation to sulfite. Joyce *et al.*[34] use MOPS-free M9 buffer and consequently shows *cysADQUW* essentiality for sulfate uptake. The MOPS desulfonation explanation of the data of [32] is complicated by the fact that sulfite from MOPS should bypass*cysNDCH* within the assimilatory sulfate reduction pathway. Instead, these deletions fall within or near the broad essentiality criteria of [5]; *cysN* is barely within the broad essentiality criteria (OD600 0.088 at 24 hr) and *cysD* is just above (OD600 0.104 at 24 hr) while *cysCH* are deep within the cutoff. We propose an explanation based on inactivation of the *ssu* pathway transcriptional regulator Cbl via binding of adenosine 5’-phosphosulfate (APS), the product of the CysND sulfate adenylyltransferase [37,38]. *cysND* mutants express the *ssu* pathway well and are able to better support growth via MOPS catabolism. *cysCH* mutants can produce APS from extracellular sulfate, leading to Cbl inactivation by APS binding, repression of *ssu*, and weaker growth.
*dapF*	+	–	+	[36,39,40]	EcoCyc lists *dapF* as the only enzyme capable of converting LL-diaminopimelate to*meso*-diaminopimelate in lysine biosynthesis, but [39] demonstrates that *dapF* null mutants grow in unsupplemented glucose minimal media. *dapF* is described in [36].
*dut*	–	+	–	[41,42]	NudI and MazG substitute for Dut activity in EcoCyc–18.0–GEM. The NudI Km for the dUTP-consuming reaction shared with Dut is in the mM range. MazG activity is 70% inhibited by the MazEF toxin-antitoxin system; see [42]. el-Hajj *et al.*[41] discuss Dut at greater length.
*folB*	+	–	N/A	[32,34,43]	*folB* reported as nonessential by [32], but essential by [34], despite no obvious reason for differential essentiality on glucose and glycerol. Haussmann *et al.*[43] studied the enzyme but did not construct a deletion mutant or test it on glucose minimal media; whether an attempt was made is unknown. *folB* is found upstream of genes reported essential by [32] in the folate biosynthesis pathway, including *folK*, *folC*, *folA*, and possibly *folP* (see below).
*folP*	–	–	N/A	[32,33,44,45]	To our knowledge, there has not been a clear *folP* or *hemE* null mutant test on glucose minimal
*hemE*	–	–	N/A		media. A *folP* deletion mutant grows poorly in rich media according to [45]. *folP* and *hemE* gene duplications preventing observation of the null phenotype in [32] were identified in [33,36] and these genes are described as of uncertain essentiality by [33]. Genes of uncertain essentiality in [33] are those with partial duplication for both isolates that are considered nonessential in [44], which tested culture growth on rich Antibiotic Medium 3 medium containing beef extract as opposed to the LB agar of [33]. Under the broad essentiality criteria of [6], genes with uncertain essentiality in [33] were considered nonessential. EcoCyc–18.0–GEM supports the conclusion of essentiality for *folP* and *hemE*.
*ftsW*	–	+	–	[46-50]	It is not clear whether FtsW, MurJ, or both carry out the lipid II flippase activity in *E. coli*. See the
*murJ*	–	+	–		listed references for additional information on this topic.
*kdsC*	–	+	+	[51-53]	*kdsC* is upstream of the essential genes *kdsB* and *waaA* in the CMP-KDO biosynthesis pathway. Sperandeo *et al.*[51] suggeststhat isozymes for KdsC’s 3-deoxy-D-*manno*-octulosonate 8-phosphate phosphatase activity may exist. Nonspecific phosphatase activity might also carry out this reaction. See [52,53] on the subject of CMP-KDO requirements and *E. coli* temperature sensitivity.
*pabC*	+	–	–	[32,34,36,54]	Green *et al.*[54] report that PABA, the product of *pabC*, is required for growth on minimal media (although the carbon source used on this media is not described) and establishes that only one copy of the gene exists in *E. coli*. PabC is upstream of enzymes reported essential by [32] in its pathway. Kim and Copley [36] hypothesize nutrient carryover from rich culture for lack of essentiality in [32]. *pabC* is also nonessential in the M9 glycerol medium of [34].

Cases where EcoCyc–18.0–GEM essentiality predictions differed from experimental gene-essentiality results for aerobic growth on MOPS medium with 0.4% glucose, and posed open biological questions or highlight metabolic network interactions of particular interest in EcoCyc–18.0–GEM. Certain of these genes deserve further investigation by the experimental community; others highlight interesting aspects of essentiality testing. Kim and Copley [36] have discussed several of these genes, which remain open issues in the literature. See text for additional details. Column headings are as follows: **HT:** High-throughput experiment (loose essentiality criteria of [6]). **Sim:** Simulation. **Conv:** Conventional experiment. Column entries are as follows: **–:** In the experiments, deletion mutant could not be recovered for testing, or was tested and did not grow to more than 0.091 OD600 after 24 hr, or both isolates were found to have duplications. In the simulations, FBA biomass flux was zero. **+:** In the high-throughput experiments, a deletion mutant was tested and showed growth greater than 0.091 OD600 after 24 hr. In the conventional experiments, growth was observed. In the simulations, FBA biomass flux positive. **N/A:** Information not available for deletion mutant on glucose minimal media. For example, *argD* is essential in the data of [32] according to the broad essentiality criteria of [6], but EcoCyc–18.0–GEM predicts that it is nonessential.

**Table 8:** False gene essentiality predictions within glycolytic and TCA cycle metabolism

**Gene**	**HT**	**Sim**	**Conv**	**Citations**	**Comments**
*aceE*	+	+	–	[32,36,55-57]	Baba et al. [32] observe *aceE* null mutant growth to OD600 0.353 (nonessential), *aceF* null mutant
*aceF*	–	+	–		growth to OD600 0.091 (the line of loose essentiality), and *lpd* null mutant growth to OD600 0.061
*lpd*	–	+	–		(loose essential). Langley and Guest [55] observe pyruvate dehydrogenase complex essentiality and [56,57] discuss it in the context of *poxAB*; [36] suggest mutants overexpressing pyruvate oxidase allow growth on glucose.
*eno*	–	+	–	[36,58,59]	Hillman and Fraenkel [58] describe *gapA* essentiality and [59] describes *eno*/*gapA*/*pgk*
*gapA*	–	+	–		essentiality. Null mutants in these enzymes suffer from glucose toxicity because of glucose
*pgk*	–	+	–		catabolite repression of other carbon utilization pathways. See also [36].
*icd*	–	+	–	[32,36,60-67]	EcoCyc–18.0–GEM *icd* deletion mutants grow via condensation of propionyl-CoA and glyoxylate to 2-hydroxyglutarate [60,61], and oxidation of 2-hydroxyglutarate to 2-oxoglutarate via LhgO [66]. *icd* is broadly essential on minimal glucose media in [32]. *icd* deletion mutants require glutamate [62,63] and can grow on LB [64,65], although [67] reports growth on M9 minimal media. See also [36].
*fbaA*	–	+	–	[36,68-71]	The class I fructose bisphosphate aldolase *fbaB* is listed as an isozyme for *fbaA* in EcoCyc, but is expressed only under gluconeogenic conditions [68,69]. Use of the *fsa*/*dhaK* pathway [70,71] to substitute for *fbaA* appears to be blocked in the results of [32] by lowered *dhaK* expression in *E. coli* with operational phosphotransferase systems. Other effects of *fbaA* deletion are discussed in [36].
*gltA*	–	+	–	[36,72,73]	PrpC, the 2-methylcitrate synthase in the propionate utilization pathway, is an isozyme for the GltA citrate synthase. *prpC* is conditionally expressed in the presence of propionate. Discussed in [36,73].
*pfkA*	–	+	–	[32,36,74,75]	*pfkA* and *pfkB* are listed as isozymes for the 6-phosphofructokinase reaction, but PfkB activity is insufficient to allow strong growth *in vivo* in the absence of *pfkA* according to [74]. *pfkA* is loosely essential (OD600 0.087) in [32]. See [36] and [75], which indicate that both *pfkA* and *pfkB* must be deleted to block growth.
*ppc*	–	+	+	[32,76,77]	Peng *et al.*[76] demonstrate growth of *ppc* deletion mutants without detectable Ppc activity on M9 glucose minimal media. *ppc* grows on rich media but is narrowly essential under minimal glucose conditions in the high-throughput assay of [32]. EcoCyc–18.0–GEM *ppc* deletion mutants grow via activation of the glyoxylate shunt, in agreement with the observations of [76]. Patrick *et al.*[77] identified overexpression of the osmoregulatory system regulator EcfM and the uncharacterized protein YccT as capable of rescuing *ppc* deletion mutants.
*tpiA*	+	+	–	[32,36,78,79]	Kim and Copley [36] describe *tpiA* essentiality based on methylglyoxal formation in *tpiA* null mutants, and the *tpiA* nonessentiality result of [32] as based on methylglyoxal pathway-expressing mutants [78,79].

The three available pathway options for navigating the route from glucose to the TCA cycle make up the superpathway of glycolysis, the pentose phosphate pathway and the Entner-Doudoroff pathway. Without modeling of regulation, product inhibition, and metabolite toxicity, the multiple re-entry points in the superpathway and the reversible nature of the pentose phosphate pathway allow carbon flux to route around deletions with weak growth. EcoCyc–18.0–GEM consequently produces several false positive results for these pathways. This set of genes has previously been discussed at length by [36]. The *aceE* and *tpiA* genes identified as essential in Figure two of [36] are judged nonessential by [32] and consequently by [6], and were predicted as nonessential by EcoCyc–18.0–GEM, but these nonessentiality conclusions appear incorrect based on conventional experiments described in the literature, and are thus included in this table. The converse is true for *ppc*, which is considered essential by high-throughput experiments, but is not essential in conventional experiment. See Table 7 caption for a description of column headings.

**Table 9:** **False gene essentiality predictions resulting from isozymes or pathways not operational under the experimental conditions of Baba *et al.***

**Gene**	**HT**	**Sim**	**Conv**	**Citations**	**Comments**
*aroE*	–	+	–	[80,81]	*ydiB* encodes an isozyme for the AroE shikimate dehydrogenase in EcoCyc. Johansson and Liden [81] suggest that the NAD ^+^/NADP ^+^ specificity of YdiB and high intracellular NAD ^+^ concentrations lead it to operate in the “reversed” shikimate dehydrogenase direction, as opposed to the biosynthetic direction toward chorismate.
*can*	–	+	–	[82]	*cynT*, a carbonic anhydrase in the *cyn* cyanate degradation operon, encodes an isozyme for the Can carbonic anhydrase reaction in EcoCyc. *cynT* is conditionally expressed in the presence of cyanate, which is absent in [32] minimal media conditions.
*folA*	–	+	–	[83,84]	*folM*, a 7,8-dihydromonapterin reductase in the tetrahydromonapterin biosynthesis pathway with a weak 7,8-dihydrofolate reductase activity, encodes an isozyme for the FolA dihydrofolate reductase reaction in EcoCyc. *folM* is insufficiently expressed in vivo to supply *E. coli* growth requirements for tetrahydrofolate.
*folD*	–	+	–	[85]	10-formyl-tetrahydrafolate formation by *folD* can be shortcircuited in EcoCyc–18.0–GEM by *purN’s* reversible phosphoribosylglycinamide formyltransferase activity. PurN’s *k*_*c* *a* *t*_ is substantially higher in the forward direction than in the reverse direction *in vitro*, but the *in vivo* reversibility of the enzyme is uncertain.
*glyA*	–	+	–	[77,86,87]	The threonine dehydrogenase Tdh and 2-amino-3-ketobutyrate CoA ligase Kbl provide an
*serA*	–	+	–		alternate, threonine-based route to glycine synthesis from serine by the GlyA serine hydroxymethyltransferase. *tdh* and *kbl* are under the control of the Lrp leucine repressor system, and are conditionally expressed in the presence of leucine, which is absent in [32] minimal media conditions. Patrick *et al.*[77] determined that overexpression of Tdh, the LtaE low-specificity threonine aldolase, the YneH glutaminase, or Rsd anti-sigma factor led to rescue of *glyA* deletion mutants, and that YneH was also capable of rescuing *serA* null mutants.
*guaB*	–	+	–	[88]	*guaB* is essential for growth on glucose in [32], and [88] indicates that *guaB* mutants require guanine for growth. In EcoCyc, nucleotide salvage pathways allow the degradation of IMP to inosine, subsequent conversion of inosine to xanthine via XapA/DeoD and XdhA, and finally conversion of xanthine to XMP via Gpt.
*ilvA*	–	+	–	[77,89]	The threonine dehydratase TdcB acts as an isozyme for the IlvA threonine deaminase in EcoCyc.*tdcB* is expressed only under anaerobic conditions. Patrick *et al.*[77] identified TdcB or EmrD multidrug efflux transporter overexpression as capable of rescuing *ilvA* deletion mutants.
*lipB*	–	+	–	[90]	The lipoyl-carrier protein N ^6^-octanoyl-L-lysine intermediate in lipoate synthesis can be produced by both LipB and LplA in EcoCyc. LplA is primarily involved in the assimilation of extracellularly sourced lipoate. See [90] for further details.
*metC*	–	+	–	[77,91-93]	MalY’s *β*-cystathionine lyase activity is listed as an isozyme for MetC in EcoCyc. *malY* is involved in complex regulatory interactions, and its expression is repressed by *malI* in WT strains. Without appropriate signaling, *malY* is not expressed. Patrick *et al.*[77] rescued *metC* deletion mutants via MalY, Alr alanine racemase, or FimE phase-variation switch regulator overexpression.
*metL*	–	+	–	[36,94]	*E. coli*’s three aspartokinases, ThrA, MetL, and LysC, are all isozymes for the aspartokinase reaction in
*thrA*	–	+	–		EcoCyc. The end-product inhibition of each of these enzymes should prevent aspartokinase gene KOs from being rescued by their isozymes, since adequate amino acid pools in the pathways of the remaining isozymes will inhibit their activity. Kim and Copley [36] suggest that *metL* is not expressed on glucose.
*nrdA*	–	+	–	[95]	The NrdDE ribonucleoside diphosphate reductase acts as an isozyme for the NrdAB ribonucleoside
*nrdB*	–	+	–		diphosphate reductase in EcoCyc. *nrdDE* is expressed only under anaerobic conditions.
*pdxB*	–	+	–	[77,96,97]	EcoCyc–18.0–GEM can overcome deletion of *serC* and *pdxB* by using the *thrB*/*ltaE* route from
*serC*	–	+	–		glycolaldehyde (produced by FolB and formed spontaneously from 3-hydroxypyruvate supplied from YeaB) for production of 4-phospho-hydroxy-threonine and subsequently pyridoxal-5’-phosphate [96,97]. Replacement of *serC* and *pdxB* by these pathways requires overexpression of *thrB* or *yeaB*/*nudL*, and growth after overexpression is reported only for solid media [97], whereas the assays of [32] were conducted in liquid media. Patrick *et al.*[77] rescued *pdxB* deletion mutants with Tdh threonine dehydrogenase or PurF amidophosphoribosyl transferase overexpression, and *serC* deletion mutants with YneH glutaminase overexpression.
*prs*	–	+	–	[32,98-100]	The PRPP biosynthesis II pathway can substitute for prs deletion in EcoCyc–18.0–GEM. This pathway is based on connection of ribose 5-phosphate through the DeoB phosphomutase to the PhnN ribose 1,5 bisphosphokinase activity via a putative ribose 1-phosphokinase activity [98,99]. PhnN is part of the *phn* operon, [99,101] whose expression is repressed under the 2 mM phosphate glucose minimal media conditions used by [100].
*serB*	–	+	–	[77,102]	EcoCyc–18.0–GEM can overcome deletion of *serB* by synthesizing serine from threonine via Tdh and reversible action of GlyA. Ravnikar and Somerville [102] isolated pseudorevertants containing elevated levels of Tdh from *ser* deletions following growth on media supplemented with threonine, leucine, arginine, lysine, and methionine followed by growth on minimal media. Patrick *et al.*[77] additionally identified overexpression of Gph, HisB, and YtjC phosphatases as capable of rescuing *serB* deletion mutants.

False KO predictions caused by the presence of isozymes that are able to catalyze the reaction in the model, but are either down-regulated under the media conditions of [32] or are for other reasons unlikely to substitute for the knocked-out enzyme in vivo. Because MetaFlux does not model regulation, it assumes that these enzymes are active. See Table 7 caption for a description of column headings.

**Table 10:** Genes for which EcoCyc–18.0–GEM predictions identified likely errors in high-throughput essentiality screening, and the EcoCyc–18.0–GEM predictions were confirmed by conventional essentiality experiments

**Gene**	**HT**	**Sim**	**Conv**	**Citations**	**Comments**
*alsK*	–	+	N/A	[32,103,104]	The circumstances leading to this gene’s essentiality in [32] are uncertain. Kim *et al.* 1997 [103] and Poulsen *et al.* 1999 [104] demonstrate that *alsK* is not required for growth on allose, and demonstrate growth on glycerol minimal media. Poulsen *et al.*[104] constructed *alsK* null mutants with transposon insertions, demonstrating that *alsK* was not required for allose catabolism, and renamed the gene *yjcT*. *alsK*/*yjcT* is listed as essential on rich media (LB) by [32].
*aroD*	+	–	–	[32,36,105,106]	Baba *et al.*[32] suggest that an *aroD* null mutant can grow on glucose minimal media, but [105] established that *E. coli* K–12 *aroD* mutants require all of the aromatic amino acids for growth. See also the discussion in [36] with reference to [106].
*atpB*	–	+	+	[73,107,108]	von Meyenburg *et al.*[107] demonstrate growth of strains lacking intact ATP synthase on
*atpC*	–	+	+		glucose and other fermentable carbon source minimal media, at reduced growth rates.
*atpE*	–	+	+		Growth of *atp* null mutants is further discussed in [73,108].
*cydA*	–	+	+	[73,109-114]	Green and Gennis [109] demonstrate aerobic growth of cyd mutants on glucose minimal
*cydC*	–	+	+		media through use of cytochrome *bo*. See references for further discussion of redundancy in *E. coli* cytochromes and operation of cytochrome *bd*-I. Discussed in [73].
*ptsH*	–	+	+	[77,115-121]	Steinsiek and Bettenbrock [120] and Escalante *et al.*[121] review glucose uptake in *E. coli*
*ptsI*	–	+	+		mutant strains with glucose PTS defects. See references for additional details. Patrick *et al.*[77] identified overexpression of FucP fucose transporter, XylE xylose transporter, or GalE UDP-glucose 4-epimerase as capable of rescuing *ptsI* deletion mutants.
*spoT*	–	+	+	[73,122-126]	*spoT*^-^ mutants grow slowly on glucose minimal media [122,123]. Absence of SpoT ppGpp hydrolase activity leads to high levels of ppGpp, which are inversely correlated with growth rate [126]. See references for additional details. Discussed in [73].
*ubiA*	–	+	+	[127,128]	Cox *et al.*[127] constructed ubiquinone-free mutants of K–12 capable of growth on
*ubiD*	–	+	+		fermentable substrates including glucose. Wu *et al.*[128] constructed *ubiA* null mutants
*ubiE*	–	+	+		capable of growing on minimal media containing fermentable carbon sources.
*waaU*	–	+	+	[129,130]	Essentiality in glucose minimal media has not been clearly determined. Klena *et al.*[129] constructed *waaU* null mutants and demonstrated their viability in rich media, in contradiction to the determination of essentiality in rich media in [32].

See Table 7 caption for a description of column headings.

**Table 11:** False gene essentiality predictions for genes representing systems beyond the scope of the EcoCyc–18.0–GEM biomass function

**Gene**	**HT**	**Sim**	**Conv**	**Citations**	**Comments**
*der*	–	+	–	[131-133]	Der is essential for maintenance of 50S ribosomal subunit stability. Der’s GTPase activity regulates the specificity of its interactions with the ribosomal subunit. Regulatory GTPase activities, such as those of Der, are beyond the current scope of EcoCyc–18.0–GEM.
*mrdA*	–	+	–	[134,135]	MrdA and FtsI, often referred to as penicillin-binding proteins 2 and 3, are essential for cell division
*ftsI*	–	+	–		and maintenance of cell shape. Ogura *et al.*[135] determined *mrdA* to be essential in K–12, while [134] determined *ftsI* to be essential. Modeling of the unique roles of *mrdA* and *ftsI* is beyond the current scope of EcoCyc–18.0–GEM.
*suhB*	–	+	–	[136]	Wang *et al.*[136] studied *suhB* null mutants, which exhibit a cold-sensitive phenotype, and reported that they were unable to grow at 30°C, but grew well at 42°C. Growth was studied at 37°C, but not described. Heat and cold sensitivity are beyond the current scope of EcoCyc–18.0–GEM.

Examples of these systems include cold response and cell envelope maintenance. The EcoCyc GEM biomass function will be expanded to incorporate the operation of these systems in future versions. See Table 7 caption for a description of column headings.

**Table 12:** False gene essentiality predictions caused by technical issues in MetaFlux and EcoCyc

**Gene**	**HT**	**Sim**	**Comments**
*bioB*	–	+	Lipoate biosynthesis and the final step of biotin biosynthesis are not operational in EcoCyc–18.0–GEM due
*birA*	–	+	to reaction mass imbalances in EcoCyc 18.0. This leads to the presence of dethiobiotin instead of biotin in
*lipA*	–	+	the objective function, and prevents biotinylation of biotin carboxyl carrier protein.
*entD*	–	+	EntDEFG activity is active in EcoCyc–18.0–GEM without EntD because of the presence of individual EntE, EntF, and EntG activity in EcoCyc in addition to an activity describing the overall EntDEFG complex, which leads to *entD* knockouts being ineffective.
*entD*	–	+	EntDEFG activity is active in EcoCyc–18.0–GEM without EntD because of the presence of individual EntE, EntF, and EntG activity in EcoCyc in addition to an activity describing the overall EntDEFG complex, which leads to *entD* knockouts being ineffective.
*fdx*	–	+	The IspG reaction in the EcoCyc MEP pathway incorrectly requires ferredoxin (encoded by *fdx*) instead of flavodoxin I [137].
*iscS*	–	+	The IscS cysteine desulfurase lacks chemical structure in EcoCyc, preventing its participation in thiazole
*thiL*	–	+	biosynthesis and iron-sulfur cluster synthesis. This affects *thiL* essentiality by blocking thiazole synthesis and rendering EcoCyc–18.0–GEM unable to synthesize thiamin. Due to exogenous thiamin contamination in the experiments of [32] (see [73]; note that *thiL* is apparently mislabeled as *thiI* therein) this omission manifests itself only as an incorrect prediction of *thiL* nonessentiality, since endogenous and exogenous thiamin pathways can substitute for knockouts in each other until the final thiamin monophosphate kinase activity of ThiL.
*metE*	–	+	MetaFlux does not correctly model polymerization reactions, which prevents the synthesis of 5-methyltetrahydropteroyl tri-L-glutamate by folate polyglutamylation. This in turn prevents MetE’s cob(I)alamin-independent methionine synthase reaction from operating properly.
*metH*	+	–	MetH’s cob(I)alamin-dependent methionine synthase activity does not currently require cob(I)alamin cofactor in EcoCyc–18.0–GEM because cofactor requirements are not accounted for in enzymatic reactions. Because MetE is inoperational as a result of a lack of folate polyglutamylation (see above), the incorrectly operational MetH methionine synthase reaction becomes essential. This ambiguity will be remedied in future versions of MetaFlux.
*ndk*	+	–	*ndk* is falsely predicted as essential because its UDP kinase and dTDP kinase activities provide the only routes in EcoCyc to UTP and dTTP, respectively. *In vivo, ndk* null mutants are rescued by broad substrate specificity of *adk*[36,138].
*pyrI*	+	–	The catalytic subunit PyrB of the aspartate transcarbamylase PyrBI is active by itself in vitro [139]. The PyrBI complex catalyzes the physiologically regulated reaction, and the reaction is assigned to the PyrBI complex in EcoCyc. This causes the *pyrI* gene KO simulation to block PyrBI aspartate transcarbamylation activity entirely.
*trxB*	+	–	The activity of the glutaredoxin pathway can substitute for the thioredoxin pathway in *E. coli*, and vice versa. This ability is not properly modeled in EcoCyc–18.0–GEM as a result of the pathways’ structure in EcoCyc.

These technical issues will be addressed in future versions of MetaFlux and EcoCyc. See Table 7 caption for a description of column headings.

Several of the false gene essentiality predictions described within these tables were discussed in the work of Kim and Copley, who examined the essentiality conclusions of Baba *et al.* in *E. coli* core metabolism with reference to the then-current state of EcoCyc. Constraint-based model improvement and gap-filling based on gene essentiality predictions derived from the work of Baba *et al.* have been examined for the COBRA family of constraint-based models of *E. coli* metabolism by Reed *et al.*[140], Kumar *et al.*[141], Kumar and Maranas [142], Barua *et al.*[143], Orth and Palsson [73,144], and Tervo and Reed [145]. Our revisions of EcoCyc–18.0–GEM included manual application of a subset of GrowMatch [142] gap-filling methods, specifically resolution of false positive gene essentiality predictions associated with blocked genes and false negative results associated with secretion of metabolites. The essentiality prediction accuracy resulting from our manual curation process is similar to the accuracy resulting from applying the full GrowMatch algorithm to the iAF1260 model.

Additional file 2: Table S3 provides detailed listings of essentiality status and model predictions, including a breakdown of gene essentiality prediction status by criteria used.

### Nutrient utilization analysis

Observation of culture growth on various nutrient sources is a foundation of microbiology [146]. EcoCyc 18.0 contains information on *E. coli* respiration for 428 types of media, including 22 conventional types of minimal growth media and 383 Biolog Phenotype Microarray (PM) wells. The 383 Biolog PM media conditions represent a high-throughput method of evaluating metabolic phenotypes in culture based on a tetrazolium dye assay of cellular respiration. Each well in a Biolog 96-well PM plate contains a standard minimal media composition plus a nutrient source that is varied across the PM plate, with the element supplied by the varying nutrient source dependent on the type of Biolog PM plate in use [147-149].

We evaluated the performance of EcoCyc–18.0–GEM in predicting growth for the following available datasets: (1) aerobic *E. coli* growth on the 22 common conventional minimal growth media; (2) consensus estimates of respiration based on four different experimentalists’ measurements of aerobic Biolog 96-well plates PM1–4, representing 313 conflict-free growth observations; and (3) an anaerobic Biolog PM1 plate assay surveying carbon source utilization in the absence of oxygen, representing 96 anaerobic growth observations. Biolog PM data stored in EcoCyc measures utilization of nutrients as sources of carbon (PM1–2), nitrogen (PM3), sulfur (PM4), and phosphorus (PM4).

Conventional media compositions and growth results were drawn from the literature. Aerobic Biolog PM nutrient utilization assay results were compiled from four different datasets captured in EcoCyc: (1) from our own experiments; (2) from a dataset obtained from B. Bochner; and from the recent publications of (3) AbuOun *et al.*[150] and (4) Yoon *et al.*[151]. Anaerobic Biolog PM nutrient utilization assay results were obtained from B. Bochner. We did not include the data of Baumler *et al.* in our analysis of Biolog PM results because of variation in culture conditions and a high degree of conflict with other datasets under both aerobic and anaerobic conditions [152]. See the Methods section for additional details.

Growth on a given type of media was tested by constructing simulated MetaFlux nutrient sets corresponding to the contents of the media in question and comparing EcoCyc–18.0–GEM growth predictions with experimental growth results. Due to the absence of enterobactin iron uptake modeling in EcoCyc, Fe ^3+^ in the medium was replaced with Fe ^2+^. Anaerobic simulations were prepared identically to those performed for aerobic growth, except for the removal of oxygen from the nutrient set, inclusion of the formate-hydrogen lyase reaction, and the removal of the protoheme and pyridoxal 5’-phosphate synthesis requirement from the biomass.

Literature-based EcoCyc curation and appropriate modifications of MetaFlux metabolite sets were used to address incorrect nutrient utilization predictions. The final results for PM array validation after curation are listed in Table 13. Overall accuracy of growth prediction for aerobic Biolog PM assays was 252/313 (80.5%), with 70 assays not evaluated because of experimental conflicts (see the Methods section). Anaerobic Biolog PM assay predictions had an overall accuracy of 74/96 (77.1%). Aerobic growth tests on conventional minimal media contained in EcoCyc had an overall accuracy of 22/22 (100.0%).

**Table 13:** EcoCyc–18.0–GEM nutrient utilization prediction results

**Nutrient utilization**	**Aerobic PM**	**Anaerobic PM**	**Conventional**
**(sim/exp)**			
True positive	137	35	17
(growth/growth)			
False positive	15	14	0
(growth/no growth)			
False negative	46	8	0
(no growth/growth)			
True negative	115	39	5
(no growth/no growth)			

**Aerobic PM:** 313 absolute consensus results from Biolog Phenotype Microarray carbon, nitrogen, sulfur, and phosphorus source tests (Biolog plates PM1–4) conducted under aerobic conditions. **Anaerobic PM:** 96 Biolog Phenotype Microarray carbon source tests (Biolog plates PM1–4) conducted under anaerobic conditions. **Conventional:** 22 *E. coli* minimal growth media described in EcoCyc.

The overall accuracy of nutrient utilization prediction across all aerobic and anaerobic PM and conventional growth media is 348/431 (80.7%). Tables 14, 15, 16 and 17 provide detailed discussions of false negatives and false positives for aerobic PM assays. Tables of results for anaerobic PM assays and conventional growth media are available in Additional file 2: Tables S8 and S9, respectively.

**Table 14:** Conflicts between EcoCyc–18.0–GEM growth predictions and experimental carbon source utilization data for aerobic growth on Biolog PM plates at 37°C

**Carbon source**	**HT**	**Sim**	**Comments**
Dextrins	+	–	General dextrin uptake and catabolism via *glg* is described in EcoCyc, but the system is not applied to the dextrins in EcoCyc–18.0–GEM because of MetaFlux’s current inability to model polymerization reactions.
Lactulose	+	–	Lactulose is taken up by the MelB melibiose transporter [153], although this route of uptake is not present in EcoCyc. Lactulose is capable of inhibiting LacY transport of *o*-nitrophenyl- *β*-D-galactopyranoside [154], and can be anaerobically fermented by *E. coli*[155], but the route of catabolism is unknown.
Methyl- *α*-D-galactopyranoside	+	–	Methyl- *α*-D-galactopyranoside is taken up via the MelB melibiose transporter [153], and is capable of inhibiting LacY transport of *o*-nitrophenyl- *β*-D-galactopyranoside [154]. The route of catabolism is unknown.
Methyl- *β*-D-galactoside	+	–	Methyl- *β*-D-galactoside is taken up via MglABC transporter or MelB transporter, but the catabolic pathway is unknown. It is capable of inhibiting LacY transport of *o*-nitrophenyl- *β*-D-galactopyranoside [154], and is reported as a substrate of LacZ [156]. Inside the cell, methyl- *β*-D-galactoside is acetylated by LacA galactoside acetyltransferase [157], after which its fate is unclear.
Methyl pyruvate	+	–	Methyl pyruvate is a competitive inhibitor of the active pyruvate transport system [158]. No route of uptake for methyl pyruvate is present in EcoCyc, and the route of catabolism is unknown.
Melibionate	+	–	These compounds’ route of uptake is unknown.
1-O-methyl- *β*-D-glucuronate	+	–	
3-O- *β*-D-galactopyranosyl-D-arabinose	+	–	
Methyl D-lactate	+	–	
Mono-methyl hydrogen succinate	+	–	
L-galactono-1,4-lactone	+	–	The route of uptake is unknown; it may be catabolized via a ring opening to L-galactonate, as with D-galactono-1,4-lactone.
*Meso*-tartrate	+	–	PM experiments indicate that *meso*-tartrate can be used as a carbon source by *E. coli*, in contradiction of the reports of [159] and [160]. *meso*-tartrate is not associated with L/D-tartrate uptake processes in EcoCyc.
Bromosuccinate	+	–	Bromosuccinate is described in the literature is as an irreversible inhibitor of aspartate transcarbamylase [161] and it may be taken up via the same pathways as aspartate. No route of uptake is present in EcoCyc.
2-hydroxybutyrate	+	–	The route of uptake is unknown.
Citrate	–	+	Most strains of *E. coli* cannot use citrate as a carbon source under aerobic conditions because of lack of transporter expression [162]; the citrate/succinate antiporter CitT is expressed under anaerobic conditions, although a cosubstrate is still required to generate reducing power to form succinate [163]. MetaFlux does not currently model gene regulation.
Putrescine	–	+	These nitrogenous compounds cannot be used as carbon sources under the high-nitrogen
4-aminobutyrate	–	+	conditions of the Biolog PM carbon source assay, given the lack of Ntr-mediated expression
Ornithine	–	+	of their catabolic pathways [164]. MetaFlux does not currently model gene regulation.
L-arginine	–	+	Arginine cannot be used as a carbon source by *E. coli* K–12 because of the absence of induction and transport [165,166]. MetaFlux does not currently model gene regulation.
Cellobiose	–	+	Cellobiose cannot be used as a carbon source by *E. coli* K–12 because of its inability to abolish repression of the ChbABC chitobiose/cellobiose PTS permease system by NagC [167,168]. MetaFlux does not currently model gene regulation.
Glycine	–	+	Biolog PM experiments employing glycine as a carbon source return a consensus no-growth result, but EcoCyc–18.0–GEM predicts that glycine can be used as a carbon source via assimilation into 5,10-methyltetrahydrofolate by the glycine cleavage system. This is a wasteful pathway, producing one CO _2_ and one molecule of 5,10-THF per glycine molecule taken up. We found no information on conventional growth experiments assaying the ability of *E. coli* K–12 to use glycine as a carbon source.
D-tartrate	–	+	D-tartrate does not support growth under aerobic conditions in the experiments of [160]. It uses the anaerobic TtdT transporter in EcoCyc–18.0–GEM; the DcuB transporter may be the correct route of entry for D-tartrate under anaerobic conditions [159].
Ethanolamine	–	+	*E. coli* requires a source of cob(I)alamin for catabolism of ethanolamine by the adenosylcobalamin-dependent ethanolamine ammonia-lyase [169-172]. MetaFlux does not currently model enzyme cofactor requirements.

Column headings are as follows: **< *Element *** **> Source:** Nutrient source under test. **HT:** High-throughput experiment. **Sim:** Simulation. Column entries are as follows: **+:** Nutrient can support growth. **–:** Nutrient cannot support growth. For example, D-fructose supports growth according to the consensus of experimental aerobic Biolog PM assays recorded in EcoCyc, but EcoCyc–18.0–GEM predicts that it does not support growth.

**Table 15:** Conflicts between EcoCyc–18.0–GEM growth predictions and experimental nitrogen source utilization data for aerobic growth on Biolog PM plates at 37°C

**Nitrogen source**	**HT**	**Sim**	**Comments**
Guanine	+	–	Guanine is not used as a nitrogen source by *E. coli*[173,174] in vivo, and its degradation does not proceed past allantoin. EcoCyc–18.0–GEM is able to use guanine as a source of ammonia by means of glucose deamination and immediate excretion of xanthine or urate.
5-aminopentanoate	+	–	Routes of uptake and catabolism are unknown for 5-aminopentanoate and glucuronamide.
Glucuronamide	+	–	
Ethanolamine	–	+	*E. coli* requires a source of cob(I)alamin for catabolism of ethanolamine by the adenosylcobalamin-dependent ethanolamine ammonia-lyase [169-172]. MetaFlux does not currently model enzyme cofactor requirements.
Allantoin	–	+	Anaerobic conditions are required for *E. coli* to use allantoin as a nitrogen source [175]. MetaFlux does not currently model gene regulation.
Nitrate	–	+	Nitrate and nitrite pass through nitrate and nitrite reductase pathways, which operate only under anaerobic
Nitrite	–	+	conditions and do not function in an assimilatory fashion in *E. coli*[176-178].
L-tyrosine	–	+	*E. coli* lacks a catabolic aromatic amino acid transaminase, preventing the utilization of L-tyrosine as a nitrogen source; the path of utilization in EcoCyc–18.0–GEM involves the tyrosine lyase used in thiazole biosynthesis followed by spontaneous dissociation of 2-iminoacetate to glyoxylate and ammonium, and is not biologically realistic due to the production of large quantities of the dead-end metabolites 5’-deoxyadenosine and *p*-cresol.

See Table 14 caption for description of column headings.

**Table 16:** Conflicts between EcoCyc–18.0–GEM growth predictions and experimental sulfur source utilization data for aerobic growth on Biolog PM plates at 37°C

**Phosphorus source**	**HT**	**Sim**	**Comments**
dTMP	+	–	dTMP, GMP, thymidine 3’-monophosphate, and thiophosphate are substrates of the periplasmic
Thiophosphate	+	–	nonspecific phosphatases PhoA and AphA, and enzymatic reactions covering these metabolites will be added in the course of EcoCyc development. Other phosphorylated metabolites have not been associated with individual phosphatases, and their pathways of utilization are a subject of future research.
3-phospho-D-glycerate	+	–	The route of phosphorus uptake from these sources is unknown. Although catabolic pathways
2-phosphoglycolate	+	–	for these phosphorylated metabolites exist in EcoCyc, the route of phosphorus uptake may
*α*-D-mannose 1-phosphate	+	–	involve nonspecific dephosphorylation.
Glucosamine-6-phosphate	+	–	
6-phospho-D-gluconate	+	–	
O-phospho-D-tyrosine	+	–	
O-phospho-D-serine	+	–	
N-phospho-L-arginine	+	–	
Trimetaphosphate	+	–	
2-deoxy-D-glucose 6-phosphate	+	–	
Creatine-phosphate	+	–	
Dithiophosphate	+	–	

See Table 14 caption for description of column headings.

**Table 17:** Conflicts between EcoCyc–18.0–GEM growth predictions and experimental sulfur source utilization data for aerobic growth on Biolog PM plates at 37°C

**Sulfur source**	**HT**	**Sim**	**Comments**
L-methionine	+	–	Methionine and related compounds enable respiration in Biolog PM assays when supplied as a sulfur
D-methionine	+	–	source. A route of catabolism is not present in EcoCyc, and methionine is not considered to support sulfur
*N*-acetyl-DL-methionine	+	–	requirements in *E. coli*[179]. This result suggests further investigation.
Gly-Met	+	–	
L-cystathionine	+	–	
L-methionine *S*-oxide	+	–	
L-cysteine	+	–	L/D-cysteine lack clear pathways of uptake in EcoCyc–18.0–GEM. This problem will be a subject of future
D-cysteine	+	–	EcoCyc development.
Thiophosphate	+	–	PhoA activity on thiophosphate will be added in future versions of EcoCyc.
Djenkolate	+	–	The route of uptake is unknown. Catabolism may proceed via MetC [180].
Lanthionine	+	–	
3-sulfinoalanine	+	–	The route of uptake is unknown. SufS and CsdA can convert 3-sulfinoalanine to alanine and sulfite.
Cysteamine	+	–	Routes of uptake and catabolism for cysteamine, dithiophosphate, hypotaurine, tetrathionate, and
Dithiophosphate	+	–	thiourea are unknown.
Hypotaurine	+	–	
Tetrathionate	+	–	
Thiourea	+	–	

See Table 14 caption for description of column headings.

### Model readability and accessibility

Scientists naturally need to ask many questions of a metabolic model, such as “What are the chemical structures of all substrates in reaction X, and is X chemically balanced?” “What metabolic pathway(s) is reaction X a member of, and what are the adjacent reactions?” “Which *E. coli* enzymes are inhibited by ADP?” “What transcriptional regulators affect the expression of the enzymes for reaction X?” Their ability to answer these questions rapidly and accurately is strongly dependent on the model representation, the software tools available for querying and visualizing that representation, the tightness with which those tools are integrated with the model, and the presence of additional enriching information for the model.

Existing *E. coli* models are represented as spreadsheet files and as SBML files, making it tedious or impossible for non-programmers to answer the preceding questions directly from those files. Although SBML files can be imported into software tools such as the RAVEN Toolbox [181] and rbionet [182], in practice that approach is limited because of variations in SBML encodings, the effort required to install and integrate multiple software tools with disparate capabilities, and the limited visualization capabilities of those tools. More fundamentally, previous *E. coli* models do not capture (nor can SBML capture) additional enriching information that, while not required for the mathematical operation of a model, greatly enhances our ability to validate and understand a model, and to answer the preceding questions. Examples of such enriching information present in EcoCyc–18.0–GEM are metabolite chemical structures, arrangements of reactions within metabolic pathways, and gene regulatory information. Note that introducing ad-hoc definitions of these data (e.g., pathways) in the SBML “Notes” field, or introducing SBML links to external databases, would be considered out of bounds: since pathways are not captured formally in the SBML specification today, there is no guarantee regarding interoperability of software tools with such ad-hoc data.

EcoCyc–18.0–GEM is highly understandable because it can be interactively queried and visualized through the EcoCyc web site and desktop Pathway Tools software, which supports visualization of metabolic pathways and reaction diagrams; metabolite pages that depict metabolite structures and all reactions a metabolite is involved in; depiction of gene/reaction connections and of genome organization via a genome browser; navigation through the *E. coli* gene regulatory network; constructing structured queries such as: find all reactions of a given metabolite; find all enzymes utilizing a given cofactor; and presentation of text summaries and citations that explain and support aspects of the model. In general, other tools for metabolic model visualization tend to be less comprehensive, and to be less closely coupled to the model; see [183-186] for recent reviews.

The reaction fluxes computed from EcoCyc–18.0–GEM are more understandable than those from previous *E. coli* models because EcoCyc–18.0–GEM fluxes can be immediately painted onto the EcoCyc Cellular Overview, a zoomable diagram of the complete metabolic map of *E. coli* that allows immediate visual inspection of flux patterns. Although other software tools exist for visualizing flux patterns on metabolic networks, e.g., the RAVEN Toolbox, they are unlikely to be easily usable with previous *E. coli* models. For example, RAVEN Toolbox requires that the user manually construct the metabolic network diagram, which could take days or weeks of effort. In contrast, Pathway Tools generates metabolic map diagrams algorithmically from a PGDB.

## Conclusions

EcoCyc–18.0–GEM demonstrates the advantages of literate modeling based on comprehensive organism databases. It provides comprehensive genome-scale coverage of the *E. coli* metabolic network, representing gene function with an unprecedented degree of accuracy.

Integration of EcoCyc–18.0–GEM into the EcoCyc database gives investigators working with the model access to the full Pathway Tools bioinformatics and data visualization suite. This allows construction of complex database queries involving the full range of biochemical entities within *E. coli*, and visualization of pathways and reactions within the model as they change throughout the course of construction. As part of EcoCyc, EcoCyc–18.0–GEM will receive frequent updates to remain abreast of recent research developments.

The process of EcoCyc–18.0–GEM construction and validation resulted in more than 80 updates to EcoCyc. These included expansion and revision of periplasmic phosphatase activities many updates to sugar transport and phosphotransferase system modeling; correction of incorrect compartment assignments; fixes for L-lactate dehydrogenase action; revisions to glutathione hydrolysis; new transport reactions for compounds identified as nutrient sources during Biolog PM testing; addition of MOPS catabolism via the alkanesulfonate pathway; removal of several incorrect reactions and gene-protein relationships; numerous fixes to reaction reversibility and directionality; several compound class reassignments to correct issues with reaction instantiation; mass rebalancing for several reactions; and revisions to ATP synthase proton stoichiometry. These updates are outlined in Additional file 2: Table S11.

The MetaFlux software has also been improved as a result of the FBA validation of EcoCyc. These improvements include upgrades to compartmentalization handling, gene deletion code, and electron transfer reaction handling. MetaFlux solution and log files have been updated to contain additional statistical information and provide a more detailed explanation of the metabolic network construction process to the user. Numerous updates and revisions to the MetaFlux model of *E. coli* have been introduced as part of this effort. Biomass metabolite sets have been revised to reflect the work of Orth *et al.*, and additional updates have been made on the basis of the validation process in order to create the most accurate final product possible.

Many questions of interest to *E. coli* modelers and experimentalists were raised in the course of EcoCyc–18.0–GEM development. By highlighting these questions and presenting them within the context of EcoCyc as a reference database, we address the interests of the general metabolic modeling audience and of *E. coli* experimentalists interested in using models to explore their results and generate new leads for research. We summarize these questions here.

Experimental measurements of respiratory fluxes in glucose-fed aerobic chemostat culture are higher than those predicted by simulation, and small quantities of succinate and lactate are generated in experimental anaerobic fermentations, suggesting interesting *in vivo* deviations from theoretical *in silico* optimality.

A small number of metabolic byproducts must be removed directly from the cytosol in the secretion set because of a lack of known salvage or excretion pathways. The fate of these metabolites is of interest.

Several incorrect essentiality predictions are associated with unclear cellular biomass requirements, pathways with potential alternate routes of catalysis, uncertain determinations of essentiality on glucose minimal media, and ambiguous or missing gene function. Similarly, nutrient utilization predictions have identified a number of compounds that lack clear pathways of entry into metabolism but are capable of supporting respiration and/or growth. Resolution of these uncertainties would improve our understanding of *E. coli* function in varying environments.

## Methods

The data used for this project were obtained from EcoCyc version 18.0, and the bioinformatics and flux analysis procedures documented here were performed in either the Web or desktop environment of the Pathway Tools 18.0 software. Pathway Tools can be downloaded, along with documentation and example files, at http://brg.ai.sri.com/ptools/. The simulation tests were constructed by using Lisp scripting and the Pathway Tools Lisp API, documented at http://brg.ai.sri.com/ptools/api/. Further details on the construction of EcoCyc–18.0–GEM can be found within Additional file 1.

EcoCyc–18.0–GEM development employed the MetaFlux component of Pathway Tools, documented in the Pathway Tools User’s Guide and in [15]. All simulations were run in solving mode on a 2.7 GHz i7 MacBook Pro with 16 GB RAM. The “minimize-fluxes: yes” option was used for taxicab norm minimization of fluxes. For additional information, see the Pathway Tools User’s Guide. A MetaFlux.fba file demonstrating simulation of aerobic growth of *E. coli* BW25113 on glucose is included as Additional file 3.

We note that the choice of stoichiometric representation of equations can affect flux balance solutions when minimization of summed flux is used as part of the objective function; see [187] for further details. Stoichiometric coefficients within EcoCyc–18.0–GEM are scaled so as to provide minimum whole-integer stoichiometry. Application of different scaling within the flux network may lead to altered flux solutions.

Briefly, MetaFlux creates a stoichiometric metabolic flux network at run time from the metabolites and reactions contained in a Pathway Tools PGDB. During network construction, MetaFlux removes PGDB reactions that are: ambiguously instantiated (see below); unbalanced or having an undetermined balance state; disconnected from the network; marked as physiologically irrelevant; involved in polymerization; involved in polymer segment or protein modification; lacking substrates on one side; containing substrate entities that are described only by strings; possessed of variable stoichiometry; or have more than 10,000 permutations that must be checked during instantiation. MetaFlux then instantiates reactions containing compound classes, replacing the class reactions with mass-balanced reactions containing instances of the relevant compound classes. The resulting set of metabolites and reactions constitutes the metabolic flux network operated on by MetaFlux. Enzymatic reaction and gene-protein relationship data encoded in the PGDB are used to associate the reactions of the network with enzymes and genes as appropriate.

COBRA [20,188] simulations of the iJO1366 *E. coli* genome-scale reconstruction were employed in order to validate the EcoCyc FBA simulations and to provide a point of comparison to existing reference models. The iJO1366 simulations were performed using the COBRA Toolbox 5.0.0 within MATLAB R2010a and cobrapy 0.2 within Python 2.7.6. All iJO1366 simulations used taxicab norm minimization of fluxes as described in the documentation for the optimizeCbModel function.

Biomass metabolite sets were constructed using the wild-type and core biomass sets of Orth *et al.* and modified according to our research findings and experimental data on gene essentiality. The ATP turnover requirement for growth-associated maintenance costs (GAM) was set to 53.95 mmol ATP/gCDW, while the ATP turnover requirement for non-growth-associated maintenance costs (NGAM) was set to 3.15 mmol ATP/gCDW, per Orth *et al.*

All coefficients of the biomass metabolite set represent millimoles (mmol) of each metabolite required per gram of cell dry weight (gCDW). Coefficients of the nutrient and metabolite sets represent specific uptake and output fluxes, in millimoles of each metabolite supplied per gram of cell dry weight per hour (mmol/gCDW/hr). An overall biomass flux of 1.0 thus represents a specific growth rate *μ* of 1.0 hr ^-1^ (one new gram of cell dry weight per gram of cell dry weight per hour).

Aerobic glucose chemostat data for experimental comparisons were obtained from [23]. Anaerobic glucose chemostat data were obtained from [24] via [25].

The protocols followed in our PM experiments are as follows: PM plates 1–4 containing 190 sole carbon sources, 95 sole nitrogen sources, 59 sole phosphate sources and 35 sole sulfur sources were used in this analysis. *E. coli* MG1655 was obtained from the Yale Coli Genetic Stock Center, pre-grown on nutrient agar and used to inoculate the plates following Biolog instructions. The data were collected and analyzed using the OmniLogH PM system, which records the color change every 15 min for each well in the 96 well assay plates. All incubations were performed at 37°C over 48 hr. For the complete details of all PM assay conditions, please refer to the original publications.

The PM nutrient-source assay is an assay of respiration based on the generation of NADH by carbon metabolism and the subsequent reduction of a tetrazolium redox dye by NADH. As such, it does not directly measure either the cell growth simulated by FBA biomass objectives or the uptake of noncarbon sources. However, checkpoint linkage of carbon-source catabolism to nitrogen, phosphorus, and sulfur source catabolism enables the tetrazolium redox dye assay to probe the metabolism of non-carbon sources [189]. Bochner describes the phenomenon of checkpoint linkage as starvation for elemental nutrient source leading to arrest of cellular respiration, due to redox imbalance or alarmone synthesis. We therefore compared PM respiration results directly with FBA growth simulation.

Biolog PM results are stored within the EcoCyc database and are accessible via the Pathway Tools API and EcoCyc website. Individual Biolog PM assay scores for each well were compared across experimental datasets to establish a consensus for comparison with EcoCyc–18.0–GEM simulation. Because simulations of nutrient utilization were scored according to growth or no growth, experimental Biolog PM results indicating ‘normal’ and ‘low’ respiration were combined into a ‘positive’ result. The majority of Biolog PM tests (313/383) displayed a consensus of either respiration or no respiration across all four experimental datasets used. In 70 of 383 cases, no clear consensus could be reached. These divergent cases were omitted from the nutrient-utilization assay validation because no reliable conclusion could be reached regarding the results. See Additional file 2: Table S10 for a list of omitted PM data.

## Competing interests

DSW, IMK, and PDK receive fees from SRI International commercial licensing of Pathway Tools.

## Authors’ contributions

PDK and DSW conceived and designed the experiments. DSW constructed the model and conducted the simulations. DSW analyzed the data. IMK, AM, and DSW curated the database. DSW and PDK wrote the manuscript. AM and ITP performed phenotype microarray experiments. All authors read and approved the final manuscript.

## Supplementary Material

Additional file 1 Supplementary information text and table descriptions. Click here for file

Additional file 2 Supplementary tables (11 tables). Click here for file

Additional file 3 **MetaFlux .fba file demonstrating simulation of aerobic growth of *E. coli *** **BW25113 on glucose.** Click here for file
