# Supplementary material for: A genome-scale metabolic flux model of Escherichia coli K–12 derived from the EcoCyc database
Source: BMC Syst Biol. 2014 Jun 30;8:79. doi: 10.1186/1752-0509-8-79 (PMC4086706; doi:10.1186/1752-0509-8-79)
Supplement: Additional file 1 — Supplementary information text and table descriptions. [file 1752-0509-8-79-S1.pdf]

Contents

1 Supplementary Tables **1**

2 Supplementary Files **2**

3 Differences between the iJO1366 biomass reaction and the EcoCyc–18.0–GEM biomass metabolite set **2**

4 The EcoCyc–18.0–GEM secretion metabolite set **3**

5 Gene essentiality definitions and BW25113 simulation **3**

6 Reactions removed in EcoCyc–18.0–GEM FBA simulations **4**

7 Genes that are not present in EcoCyc–18.0–GEM which are associated with reactions in EcoCyc **4**

8 Genes present in the iJO1366 model that are not present in EcoCyc–18.0–GEM **4**

1 Supplementary Tables

The Supplementary Tables are included as sheets of the Excel workbook `suppl-info.xlsx`.

Supplementary Table 1 All elements of the EcoCyc–18.0–GEM biomass metabolite sets, minimal media nutrient sets, and secretion set.

Supplementary Table 2 Complete side-by-side comparison of the EcoCyc–18.0–GEM and iJO1366 core and expanded biomass metabolite sets.

Supplementary Table 3 Detailed listings of experimental and simulation gene essentiality status and model predictions, including a breakdown of gene essentiality prediction status by criteria used.

Supplementary Table 4 Genes with associated enzymatic reactions in EcoCyc that are not part of EcoCyc–18.0–GEM.

Supplementary Table 5 Genes present in the iJO1366 model that are not present in EcoCyc–18.0–GEM.

Supplementary Table 6 Genes present in EcoCyc–18.0–GEM that are not present in iJO1366.

Supplementary Table 7 Detailed listings of experimental and simulation nutrient utilization status and model predictions for aerobic Biolog PM assays.

Supplementary Table 8 Detailed listings of experimental and simulation nutrient utilization status and model predictions for anaerobic Biolog PM assays.

Supplementary Table 9 Detailed listings of experimental and simulation nutrient utilization status and model predictions for aerobic conventional growth media.

Supplementary Table 10 Experimentally conflicted aerobic Biolog PM assays.

Supplementary Table 11 Curations to EcoCyc resulting from EcoCyc-18.0-GEM construction.

2 Supplementary Files

A MetaFlux input file demonstrating simulation of *E. coli* growth on glucose under aerobic conditions is included as `GlucoseAer.fba`.

3 Differences between the iJO1366 biomass reaction and the EcoCyc–18.0–GEM biomass metabolite set

We group all iJO1366 biomass fluxes to linked disaccharide tri- and tetrapeptide murein units (murein3p3p[p] (a syntax indicating two linked disaccharide tripeptide murein units (uncrosslinked, middle of chain); see supplementary information of Orth et al. for further details), murein3px4p[p], murein4p4p[p], murein4px4p[p], and murein4px4px4p[p]) under one flux to lipid II (C6[CCO-CYTOSOL]). We do not detail the process of crosslink formation in murein units or include the cost of crosslinking in synthesis.

We group all iJO1366 biomass fluxes to cardiolipins (clpn160, clpn161, and clpn181) under one flux to cardiolipin (CPD-12824); all iJO1366 biomass fluxes to phosphatidylethanolamines (pe160, pe161, and pe181) under one flux to 1,2- dipalmitoyl-phosphatidyl-ethanol (CPD-12819); and all iJO1366 biomass fluxes to phosphatidylglycerols (pg160, pg161, and pg181) under one flux to 1,2-dipalmitoyl- phosphatidylglycerol (CPD-8260). Export to periplasm is not modeled. Variation in fatty acid moieties is not modeled.

We group Fe^{2+} and Fe^{3+} requirements into a single Fe^{2+} requirement due to work in progress on the Fe^{3+} iron uptake system. Enterobactin is modeled, but enterobactin's role in iron uptake is not modeled. Ferric iron is currently replaced with ferrous iron in our simulations.

We do not currently include the following ions and inorganic cofactors in our biomass model: iron-sulfur clusters, Cu^{2+}, MoO_4^{2-}, (2fe2s[c], 4fe4s[c], cu2[c], mobd[c]). We do not yet model all metal ion uptake pathways.

We do not currently include the following organic cofactors in our biomass models: bis-molybdopterin guanine dinucleotide; molybdopterin cytosine dinucleotide; molybdopterin guanine dinucleotide; adenosylcobalamin; and thiamine diphosphate (bmocogdp[c], mococdp[c], mocogdp[c], adocbl[c], and thmpp[c]). Although some protein-bound cofactors and reaction participants are modeled in EcoCyc–18.0–GEM, these cofactors are not currently covered.

We do not currently include glycogen (glycogen[c]) manufacture in our biomass sets. Biopolymer creation and degradation is not currently implemented in EcoCyc–18.0–GEM, and polymerization reactions do not function.

Based on experimental gene essentiality analysis of EcoCyc–18.0–GEM, we have added small metabolite requirements, and compensatory precursor metabolite terms where needed, for cardiolipin, palmitate (as palmitoleoyl-ACP), lipoate, biotinylated biotin carboxyl carrier protein monomers, deformylated peptide N-terminal methionine, lysidine34 in tRNAIle2,inosine34 in tRNAArg2, N1-methylguanine37 in tRNA, 2'-O-methylcytidine1402 in 16S rRNA, N-formyl-L-methionyl-tRNAfmet, and [release factor]-N5-methyl-L-glutamine.

These small requirements are intended to force the model to generate these required precursors while presenting a minimal quantitative burden to overall metabolism. See the work of Beste et al. on *Mycobacterium tuberculosis* for further discussion of this technique.

Based on experimental gene essentiality analysis of EcoCyc–18.0–GEM in the ubiquinol-8 synthesis pathway, we have replaced the requirement for 2-octaprenylphenol in the core metabolite set with a requirement for octaprenyl diphosphate. This reflects the unambiguously established essentiality of *ispB* [1], and avoids an *ubiC* false negative and *ubiD* false positive result (the latter due to *ubiX* action as an isozyme in EcoCyc) at the cost of *ubiA* and *ubiE* false positives.

Inspired by the work of Aung et al. and previous authors on *S. cerevisiae*, we have added modeling of tRNA charging to the biomass metabolite set, along with compensatory ATP/AMP fluxes in order to maintain the originally modeled ATP maintenance demand.

Biotin is replaced with its precursor dethiobiotin in the EcoCyc–18.0–GEM biomass function due to mass imbalance in the BioB biotin synthase reaction in EcoCyc 18.0. The operation of LipA lipoate synthase is similarly blocked by mass imbalance. Lack of biotin synthase activity in turn blocks the operation of the

BirA biotin ligase in EcoCyc–18.0–GEM. This results in false positive gene essentiality results for *bioB*, *lipA*, and *birA*.

The EcoCyc–18.0–GEM reaction network currently requires oxygen to synthesize protoheme and pyridoxal-5'-phosphate. These components are removed from the biomass function under anaerobic conditions.

4 The EcoCyc–18.0–GEM secretion metabolite set

The secretion set includes the gaseous products CO_2, O_2, H_2, and CO. Water and protons are included as possible secretions, as are the mixed acid fermentation products formate, acetate, succinate, D-lactate, and ethanol. Phosphate is included in order to reflect cases of dephosphorylation of phosphorylated compounds prior to their use as nutrient sources.

All 20 amino acids are included as secretions; several of these amino acids, such as serine and valine, lead experimentally to growth inhibition when present in culture media due to regulatory effects outside the scope of EcoCyc–18.0–GEM. Purine and pyrimidine nucleobases commonly observed as degradation products in *E. coli* are present in the secretion set, including uracil, xanthine, hypoxanthine, and thymine. Urea is included due to its excretion as part of allantoin and arginine degradation pathways. Pyruvate is also included, as its secretion has been observed in *E. coli* [2]. High levels of secretion of this important metabolic intermediate should be treated skeptically, since this indicates a high degree of energy spillage in the simulation.

Several compounds produced as minor byproducts of metabolism round out the secretions set. Compounds produced outside the cytosol or possessing routes of secretion from the cytosol include D-alanine and undecaprenyl diphosphate, produced in the course of peptidoglycan dimer formation; S-methyl-5-thio-D-ribose [3], produced during spermidine biosynthesis; 4-methylphenol [4], produced during thiazole biosynthesis; two types of *myo*-inositol pentakisphosphates produced by dephosphorylation of phytate, methanol produced during biotin synthesis; AI-2, which is produced from *S*-adenosyl-L-homocysteine; betaine, which is produced in the course of choline metabolism; the glucopyranose class, which is produced by the degradation of a-D-glucose 1-phosphate in EcoCyc; and finally cysteamine, which is produced from the nonspecific dephosphorylation of cysteamine *S*-phosphate.

A dead-end metabolite (DEM) is a metabolite produced by the metabolic reactions of an organism that has no reactions consuming it, or consumed by the metabolic reactions of an organism that has no reactions producing it, and has no identified transporter [5]. 5-deoxy-D-ribose, a product of thiazole biosynthesis, is an example. In EcoCyc–18.0–GEM, DEMs are "secreted" directly from the cell cytoplasm in order to allow essential reactions to proceed without blocks from an unsecreted, unmetabolized terminal metabolite. FBA solutions containing high rates of production of DEMs should be considered dubious.

DEMs included in the secretion set and removed directly from the cytosol include 5-deoxy-D-ribose, produced in biotin and THF synthesis; 5'-deoxyadenosine, normally degraded to 5-deoxy-D-ribose by the product of the nonessential gene *mtn*; aminoacetaldehyde, produced by taurine degradation; S-adenosyl-4-methylthio-2-oxobutanoate, produced by biotin synthesis; butanal, produced by 1-butanesulfonate degradation; glycolaldehyde, produced during THF synthesis; and 3-(N-morpholino)propanesulfonate, produced during breakdown of MOPS. Glycolaldehyde has existing degradation pathways, but the action of these pathways as a result of THF synthesis lead to false negative gene essentiality predictions for *aldA* and *glcDEF*.

5 Gene essentiality definitions and BW25113 simulation

Experimental gene knockout essentiality information was drawn from [6] as emended by [7] for glucose and from [8] for glycerol, via Supplementary Table 13 of [9]. *coaA* (b3974) was added to the [9] list of always-essential genes due to its inclusion in [7], while *ptsA* (b3947) and *tnaB* (b3709) were removed from this dataset, based on the data of [6] Broad essentiality was defined according to the standard of [9]. Narrow essentiality was defined as OD600 $\leq$ 0.005 at 24 and 48 hours in [6] for glucose and as the list of genes deemed essential on rich media for glycerol.

Experimental essentiality screens carried out by [6], [7] and [8] employed the *E. coli* BW25113 strain. *E. coli* BW25113 is a derivative of *E. coli* K–12 MG1655 lacking the araBAD, rhaBAD, and lacZ genes. Since the EcoCyc database is based on *E. coli* K–12 MG1655, these genes were computationally knocked out as

described above prior to conducting the essentiality screen. EcoCyc reactions removed by these knockouts include: RHAMNULOKIN-RXN (rhaB); RHAMNISOM-RXN and LYXISOM-RXN (rhaA); RHAMNULPALDOL-RXN (rhaD); RXN0-5116 (araB); ARABISOM-RXN (araA); RIBULPEPIM-RXN (araD); RXN0-5363 and BETAGALACTOSID-RXN (lacZ).

6 Reactions removed in EcoCyc–18.0–GEM FBA simulations

In order to achieve more accurate flux distributions in the absence of detailed modeling of regulation, four reactions are removed from EcoCyc–18.0–GEM simulations depending on the circumstances of our simulation. Under circumstances of aerobic growth, the formate-hydrogen lyase reaction (FHLMULTI-RXN) is removed in order to prevent inaccurate secretion of CO_2 and H_2. Under circumstances not involving growth on glucosamine and GlcNAc, the NagB glucosamine 6-phosphate deaminase reaction (GLUCOSAMINE-6-P-DEAMIN-RXN) is disabled to avoid a futile cycle between NagB and GlmS. This removal leads to a true prediction of essentiality (true negative) for *glmS* and *glnA* during growth on glucose. *In vivo*, *nagB* is repressed under normal conditions. Addition of GLUCOSAMINE-6-P-DEAMIN-RXN to the model will result in a false prediction of nonessentiality (false positive) for *glmS* and *glnA*. Finally, we remove the MalX permease reactions TRANS-RXN0-574 and TRANS-RXN0-575, since MalX is not constitutively expressed and these reactions tend to replace the operation of the phosphotransferase system due to flux minimization.

Seven reactions in EcoCyc are removed from our model in all cases due to incorrect reversibility and/or creation of biochemically dubious pathways. These are RXN0-6359, which leads to hydrogen cyanide production; R15-RXN, which allows promiscuous transamination; MCPMETEST-RXN, which spuriously produces methanol; RXN0-6945, which allows a dubious cycle involving mercaptopyruvate; RXN-3521 and RXN0-5364, which both lead to hydrogen peroxide production. The lipopolysaccharide transport reaction TRANS-RXN0-531 is removed from the model because it provides a redundant pathway to TRANS-RXN-237 for lipopolysaccharide export but does not fully model gene requirements, leading to incorrect essentiality predictions.

7 Genes that are not present in EcoCyc–18.0–GEM which are associated with reactions in EcoCyc

Reactions in EcoCyc may be automatically excluded from EcoCyc–18.0–GEM for several reasons. These include: reaction instantiation failure due to compound substitution ambiguities; instantiated reactions with more than 10,000 instance substitutions; reactions with indeterminate or incorrect mass balance; reactions that cannot be reached from the nutrient set by any path; reactions curated as not physiologically relevant; polymerization reactions; some reactions modifying polymer segments and proteins; transport reactions transporting only protons; reactions lacking substrates on one or both sides; reactions containing substrates described only as strings; and reactions containing substrates with variable stoichiometries. Genes coding for these reactions are listed in Supplementary Table 4. Of the 328 genes coding for these reactions, 25 are experimentally essential on glucose minimal media.

8 Genes present in the iJO1366 model that are not present in EcoCyc–18.0–GEM

Differences in scope and content exist between the iJO1366 model and EcoCyc–18.0–GEM. 168 genes are assigned to reactions within iJO1366 but are not present in EcoCyc–18.0–GEM, and are listed in Supplementary Table 5; similarly, 247 genes with reactions in EcoCyc–18.0–GEM are not present in iJO1366, and are listed in Supplementary Table 6. 6 of the 168 genes present in iJO1366 but not present in EcoCyc–18.0–GEM are experimentally essential: *ubiB, wzyE, acpS, ligA, fldA*, and *lnt*. 11 of the 247 genes present in EcoCyc–18.0–GEM but not present in iJO1366 are experimentally essential on glucose minimal media (*tilS, rsmI, def, trmD, der, prmC, tadA, birA, ftsW, lptE, lptD*) and of these all but *der*, *birA* and *ftsW* are correctly predicted by EcoCyc–18.0–GEM.

References

[1] K. Okada, M. Minehira, X. Zhu, K. Suzuki, T. Nakagawa, H. Matsuda, and M. Kawamukai. The *ispB* gene encoding octaprenyl diphosphate synthase is essential for growth of *Escherichia coli*. *J Bacteriol*, 179(9):3058–60, 1997.

[2] J. Kreth, J. W. Lengeler, and K. Jahreis. Characterization of pyruvate uptake in *Escherichia coli* K–12. *PLoS One*, 8(6):e67125, 2013.

[3] J. A. Hughes. In vivo hydrolysis of S-adenosyl-L-methionine in *Escherichia coli* increases export of 5-methylthioribose. *Can J Microbiol*, 52(6):599–602, 2006.

[4] M. Kriek, F. Martins, M. R. Challand, A. Croft, and P. L. Roach. Thiamine biosynthesis in *Escherichia coli*: identification of the intermediate and by-product derived from tyrosine. *Angew Chem Int Ed Engl*, 46(48):9223–6, 2007.

[5] A. Mackie, I.M. Keseler, L. Nolan, P.D. Karp, and I.T. Paulsen. Dead end metabolites — defining the known unknowns of the *E. coli* metabolic network. *PLoS One*, 8(9):e75210, 2013.

[6] T. Baba, T. Ara, M. Hasegawa, Y. Takai, Y. Okumura, M. Baba, K. A. Datsenko, M. Tomita, B. L. Wanner, and H. Mori. Construction of *Escherichia coli* K–12 in-frame, single-gene knockout mutants: The Keio collection. *Mol Syst Biol*, 2:2006.0008, 2006.

[7] N. Yamamoto, K. Nakahigashi, T. Nakamichi, M. Yoshino, Y. Takai, Y. Touda, A. Furubayashi, S. Kinjyo, H. Dose, M. Hasegawa, K. A. Datsenko, T. Nakayashiki, M. Tomita, B. L. Wanner, and H. Mori. Update on the collection of *Escherichia coli* single-gene deletion mutants. *Mol Syst Biol*, 5:335, 2009.

[8] A. R. Joyce, J. L. Reed, A. White, R. Edwards, A. Osterman, T. Baba, H. Mori, S. A. Lesely, B. Ø. Palsson, and S. Agarwalla. Experimental and computational assessment of conditionally essential genes in *Escherichia coli*. *J Bacteriol*, 188(23):8259–8271, Dec 2006.

[9] J. D. Orth, T.M. Conrad, J. Na, J.A. Lerman, H. Nam, A.M. Feist, and B.O. Palsson. A comprehensive genome-scale reconstruction of *Escherichia coli* metabolism — 2011. *Mol Syst Biol*, 7:535, 2011.
